# MLL-AF9 regulates transcriptional initiation in mixed lineage leukemic cells

**DOI:** 10.1016/j.jbc.2024.107566

**Published:** 2024-07-11

**Authors:** Zimei Yang, Ge Zhang, Ruoyu Zhao, Tian Tian, Junhong Zhi, Gang Wei, Robert G. Roeder, Lili Jing, Ming Yu

**Affiliations:** 1 Sheng Yushou Center of Cell Biology and Immunology, School of Life Sciences and Biotechnology, Shanghai Jiao Tong University, Shanghai, China; 2 CAS Key Laboratory of Computational Biology, Shanghai Institute of Nutrition and Health, Chinese Academy of Sciences, University of Chinese Academy of Sciences, Shanghai, China; 3 Laboratory of Biochemistry and Molecular Biology, The Rockefeller University, New York, New York, USA; 4 School of Pharmacy, Shanghai Jiao Tong University, Shanghai, China

**Keywords:** mixed lineage leukemia, MLL1, MLL-AF9, super elongation complex, AF9, initiation

## Abstract

Mixed lineage leukemia–fusion proteins (MLL-FPs) are believed to maintain gene activation and induce MLL through aberrantly stimulating transcriptional elongation, but the underlying mechanisms are incompletely understood. Here, we show that both MLL1 and AF9, one of the major fusion partners of MLL1, mainly occupy promoters and distal intergenic regions, exhibiting chromatin occupancy patterns resembling that of RNA polymerase II in HEL, a human erythroleukemia cell line without *MLL1* rearrangement. MLL1 and AF9 only coregulate over a dozen genes despite of their co-occupancy on thousands of genes. They do not interact with each other, and their chromatin occupancy is also independent of each other. Moreover, AF9 deficiency in HEL cells decreases global TBP occupancy while decreases CDK9 occupancy on a small number of genes, suggesting an accessory role of AF9 in CDK9 recruitment and a possible major role in transcriptional initiation *via* initiation factor recruitment. Importantly, MLL1 and MLL-AF9 occupy promoters and distal intergenic regions, exhibiting identical chromatin occupancy patterns in MLL cells, and MLL-AF9 deficiency decreased occupancy of TBP and TFIIE on major target genes of MLL-AF9 in iMA9, a murine acute myeloid leukemia cell line inducibly expressing MLL-AF9, suggesting that it can also regulate initiation. These results suggest that there is no difference between MLL1 and MLL-AF9 with respect to location and size of occupancy sites, contrary to what people have believed, and that MLL-AF9 may also regulate transcriptional initiation in addition to widely believed elongation.

Mixed lineage leukemia (MLL) includes acute myeloid leukemia (AML), acute lymphoid leukemia (ALL), and mixed phenotype acute leukemia, occurs in children and adults, accounting for 70 to 80% of infant leukemia cases and 5 to 10% of adult acute leukemia cases and is the most common infant leukemia (1). It is caused by rearrangement of *MLL1* (MLLr), located on 11q23 and encoding one of the histone 3 lysine 4 methyltransferases in human cells. MLLr includes translocation and partial tandem duplication, and ∼90% of the MLL cases are caused by the former. Translocation of *MLL1*, that is, the break of *MLL1* and the fusion of its 5′ fragment in-frame to the 3′ fragment of one of the fusion partners mainly on other chromosomes, gives rise to an MLL fusion gene. A resulting MLL fusion protein has been known to initiate leukemogenesis through aberrantly regulating gene expression, but the molecular mechanisms are incompletely understood.

MLL1 (also known as KMT2A) is required for normal hematopoiesis (2) and is essential for embryonic hematopoietic stem cell (HSC) development (3), maintenance of quiescence of adult HSCs, and promoting proliferation of myeloid-erythroid progenitors (4). Newly translated MLL1 consists of 3969 amino acids and contains multiple motifs and domains, starting from several AT-hook motifs close to the N terminus, followed by one CXXC domain, three PHD motifs, one bromodomain, one PHD motif, one FY-rich N-terminal (FYRN) domain and one FY-rich C-terminal (FYRC) domain, and ending with one SET catalytic domain close to its C terminus (5). It is posttranslationally processed by Taspase1 between FYRN and FYRC into two fragments, the 320 kDa MLL-N and the 180 kDa MLL-C (6), which dimerize through FYRN and FYRC to form the mature MLL1. MLL1 usually forms a multiprotein complex through MLL-C with several other proteins, including WDR5, ASH2L, RBBP5, and DPY30, and they are capable of stimulating its enzymatic activity (7). In addition, MLL-N is capable of interacting with menin and lens epithelium derived growth factor (also known as PSIP1), which facilitates the recruitment of MLL1 (7). Notably, Menin inhibitors are in clinical trials for the treatment of relapsed or refractory NPM1–mutated acute leukemia and acute leukemia with MLLr and have shown on-target effects, tolerable toxicity, and promising clinical activity (1, 8).

Over 60 MLL1 fusion partners have been identified. The most common ones are AFF1 (also known as AF4), AF9, ENL, AF10, AF17, and ELL1, accounting for over 90% of the cases, and are subunits of the super elongation complex (SEC) and/or the disruptor of telomeric silencing 1-like (DOT1L) complex (9). AF9 and ENL are members of the YEATS domain–containing family (10) and are capable of participating in the SEC and the DOT1L complex in a mutually exclusive manner. In addition to AF9 or ENL, the SEC also includes AFF1 or AFF4, positive transcription elongation factor b (p-TEFb, a heterodimer of CDK9 and cyclin T1), ELL1 or ELL2, and EAF1 or 2 (11), and the DOT1L complex also includes AF10 and AF17 (12). P-TEFb plays a critical role in transcriptional elongation through releasing promoter-proximal paused RNA polymerase II (Pol II) by phosphorylating serine 2 of C-terminal domain (CTD) of Pol II, the SPT5 subunit of DSIF and NELF (13). The rest of the SEC subunits are capable of regulating pause release through P-TEFb with incompletely understood mechanisms (14). DOT1L is the only histone 3 lysine 79 (H3K79) methyltransferase in human cells and was considered an elongation factor because of the distribution of H3K79 dimethylation on and downstream of transcription start sites (TSSs) (5). Therefore, it is believed by many that major MLL-fusion proteins (FPs) maintain gene activation through aberrantly stimulating transcriptional elongation. However, we recently found that the DOT1L complex regulates transcriptional initiation in human K562 cells by facilitating the recruitment of transcription factor II D (TFIID) (15), so it would not be surprising that at least some of the major MLL-FPs are capable of aberrantly simulating initiation besides elongation. Moreover, relationship between MLL1 and its major fusion partners in transcriptional regulation in cells without MLLr is poorly understood. Related work undoubtedly would facilitate understanding molecular mechanisms underlying the aberrant gene activation maintenance by major MLL-FPs in MLLr cells.

*HOXA9* and *MEIS1* are key target genes of MLL-FPs in MLL (16). They are required for hematopoiesis (17, 18), normally highly expressed in hematopoietic stem and progenitor cells, and gradually downregulated during differentiation (19, 20). However, MLL-FPs are able to prevent the downregulation, resulting in the development of MLL (21). Analysis of chromatin occupancy of MLL1 by chromatin immunoprecipitation sequencing (ChIP-seq) found that it is mainly associated with promoters regions in human cells (22), is mainly associated with intergenic and intronic regions in murine cells (23), and contributes to the activation of a subset of genes, notably the 5′ *HOXA* genes, in histone methylation–dependent and histone methylation–independent manners (24, 25). In contrast, chromatin occupancy pattern of subunits of the SEC may be context-dependent, with one study using mouse embryonic stem cells showing that most of the SEC subunits exhibit a chromatin occupancy pattern resembling that of Pol II (26) and another study by us showing that in human THP1 cells, which express MLL-AF9 arising from a (9;11) (p22;q23) translocation, CDK9 occupies promoters and enhancers with a chromatin occupancy pattern different from that of Pol II (27). Notably, several previous studies suggested differences between MLL1 and MLL-FPs with respect to location (23) and size (22, 28, 29) of occupancy sites, which are considered to be signatures of major target genes of MLL-FPs. However, chromatin occupancy pattern of MLL1 and major MLL-FPs have never been carefully compared in the same human MLL cells before.

To further understand the molecular mechanisms of maintaining gene activation by MLL-FPs, we analyzed the chromatin occupancy of MLL1 and AF9, the relationship between their chromatin occupancy and the role of AF9 in transcriptional regulation in the non-MLLr human erythroleukemic (HEL) cells first, and analyzed the chromatin occupancy of MLL1 and MLL-AF9 and the role of MLL-AF9 in transcriptional regulation in MLL cells afterward. Our results suggest that contrary to what people have believed, there is no major difference between MLL1 and MLL-AF9 with respect to location and size of occupancy sites and that MLL-AF9 may be capable of regulating initiation in addition to transcriptional elongation.

## Results

### MLL1 mainly occupies TSS-proximal CpG islands in HEL cells

Elucidation of relationship between MLL1 and its major fusion partners in transcriptional regulation in non-MLLr cells would facilitate understanding the aberrant maintenance of gene activation by MLL-FPs in MLLr cells. AF9, the most frequent fusion partner of MLL1 in AML, is expressed at high level in HSCs and hematopoietic progenitor cells and gradually downregulated during differentiation (30). The HEL cell line was chosen for our study because of its high-level expression of AF9. To elucidate the relationship between MLL1 and AF9 in transcriptional regulation, we started by analyzing their chromatin occupancy pattern by cleavage under target and tagmentation (CUT&Tag). We also performed CUT&Tag for Pol II for determining whether MLL1 is capable of traveling with Pol II during elongation in HEL cells and further comparison of chromatin occupancy patterns of Pol II and the SEC considering that chromatin occupancy pattern of the latter may be context-dependent (26, 27).

**Figure 1:**
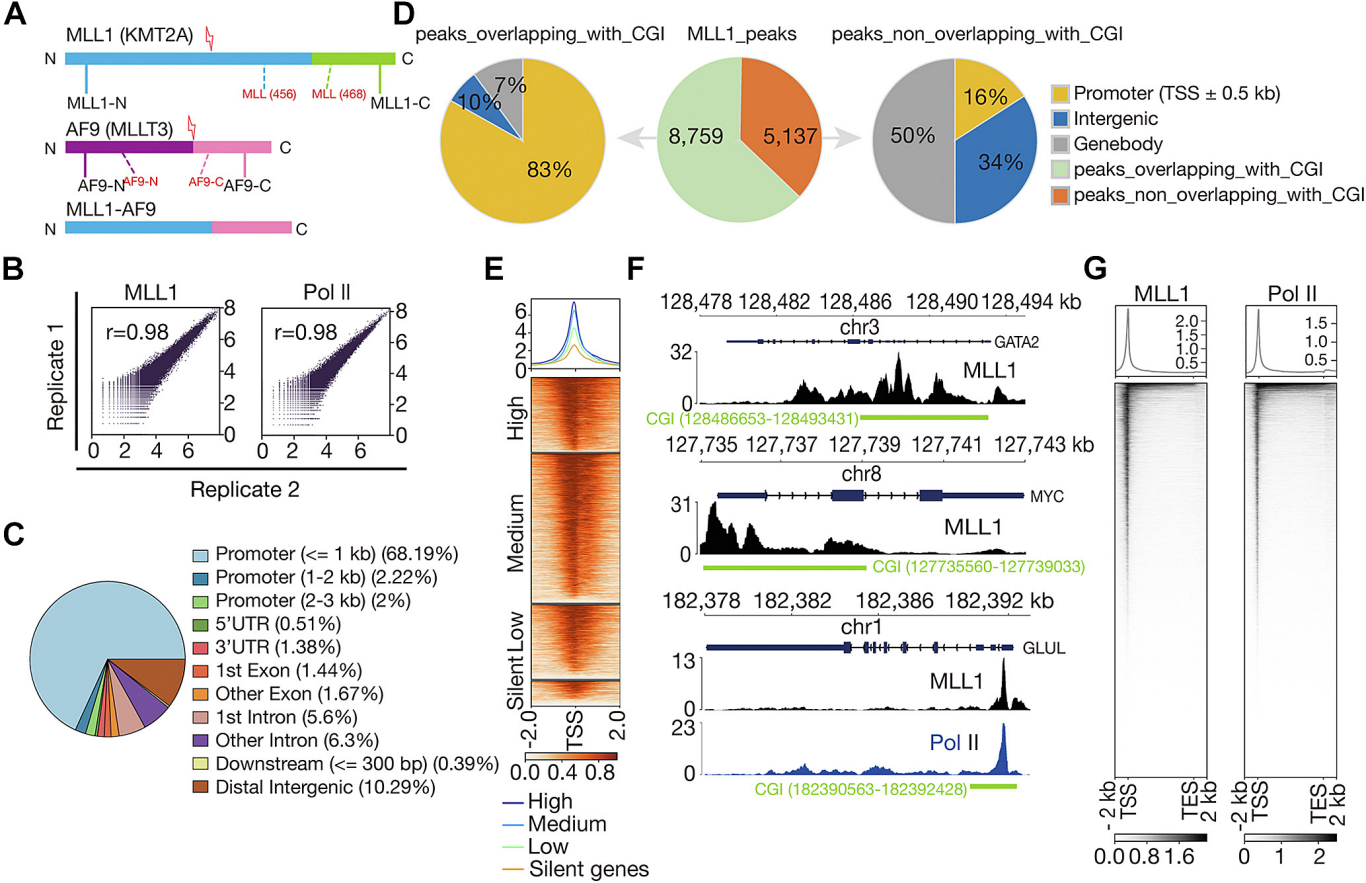
**MLL1 mainly occupies TSS-proximal CpGs in HEL cells.***A*, schematic showing MLL1, AF9, and MLL-AF9. Regions recognized by antibodies of MLL1 and AF9 used in this study are indicated with *dotted lines*. *B*, correlation plots for biological replicates of MLL1 (*left*) and Pol II (*right*) CUT&Tag experiments. Note that these CUT&Tag experiments actually had been performed in HEL cells transduced with control shRNAs, and that the plots have also been presented (reused) in Figures 4*E* and 5*A*, respectively, side by side with the related plot of knockdown cells, to make the results look complete. *C*, annotation of location of MLL1 peaks in terms of genomic features. *D*, annotation of location of MLL1 peaks in terms of CGI and TSS-proximal CGI. The *pie in the middle* shows number and percentage of MLL1 peaks overlapping with CGI and nonoverlapping with CGI, the *pie on the left* shows genomic distribution of MLL1 peaks that are overlapping with CGI, and the *pie on the right* shows genomic distribution of MLL1 peaks that are nonoverlapping with CGI. *E*, meta-gene profiles and heatmaps showing a positive correlation between MLL1 occupancy near TSSs and mRNA levels of MLL1-bound genes. Genes were sorted according to MLL1 occupancy level detected by CUT&Tag. *High:* top 25% of the nonsilent genes (mean TPM ≥ 1); *medium:* nonsilent genes between top 25% and bottom 25%; *low*: bottom 25% of the nonsilent genes; and *silent*: genes with mean 0 < TPM < 1. *F*, normalized read distribution of MLL1 within the *GATA2* (*top*) and the *c-MYC* (*middle*) loci and of MLL1 and Pol II (*bottom*) within the *GLUL* locus. *G*, genome-wide meta-gene profiles and heatmaps of MLL1 (*left*) and Pol II (*right*) CUT&Tag experiments. CGI, CpG island; CUT&Tag, cleavage under target and tagmentation; HEL, human erythroleukemic; MLL1, mixed lineage leukemia 1; Pol II, polymerase II; TPM, transcripts per million; TSS, transcription start site.

An MLL1 antibody, MLL (468) (31), against MLL-C was used for the CUT&Tag experiments of MLL1 in HEL cells (Fig. 1*A*). Correlation analyses of related biological replicates indicate that the data are highly reproducible (Fig. 1*B*). With a false discovery rate (FDR) threshold of 0.01, we identified 13,902 peaks. To determine its chromatin occupancy pattern, we analyzed overlap of peaks with known genomic features. 72.4%, 5.0%, 11.9%, and 10.3% of the peaks are overlapping with promoters, exons, introns, and distal intergenic regions, respectively (Fig. 1*C*). It is known that MLL1 is capable of binding unmethylated CpG through its CXXC domain (32), and over 50% of the promoters within the human genome are near CpG islands (CGIs) (33, 34). The finding that a high percentage of MLL1 peaks in HEL cells overlap with promoters led us to analyze overlap between MLL1 peaks and CGIs and in particular, overlap between MLL1 peaks and TSS-proximal CGIs, defined as those overlapping with regions 0.5 kb upstream and downstream of the TSSs. 8759 (63%) of the peaks were found to overlap with CGIs, and 7273 (52%) of the peaks were found to overlap with TSS-proximal CGIs (Fig. 1*D*). Together, these results suggest that MLL1 mainly occupies TSS-proximal CGIs.

Occupancy level of some factors, including Pol II, the PAF1 complex and TOX4, *etc.*, on genes were found to be positively correlated with level of mRNAs (12, 27, 35). To determine if that is also the case for MLL1, we first analyzed mRNA of HEL cells by RNA-seq, performed comparative analysis of level of MLL1 occupancy and mRNA level of genes afterward and found a positive correlation between them (Fig. 1*E*). Several previous studies suggested that major MLL-FPs, not MLL1, exhibit an extended occupancy pattern on some of the major targets of MLL-FPs (22, 28, 29). However, we found that MLL1 also exhibits an extended occupancy pattern on a subset of genes, including *GATA2* and *c-MYC* (Fig. 1*F*). After a closer examination, we found that they usually are short with TSSs and the 5′ part of their gene bodies overlapping with CGIs, where MLL1 has a high probability to occupy. Moreover, although both MLL1 and Pol II exhibit highest occupancy near TSSs (Fig. 1, *F* and *G*) and much lower occupancy downstream of TSSs, the decrease of MLL1 occupancy downstream of TSSs usually is sharper than that of Pol II occupancy (Fig. 1*F*), suggesting it unlikely for MLL1 to travel with Pol II along gene body during elongation.

### AF9 mainly occupies TSSs in HEL cells

**Figure 2:**
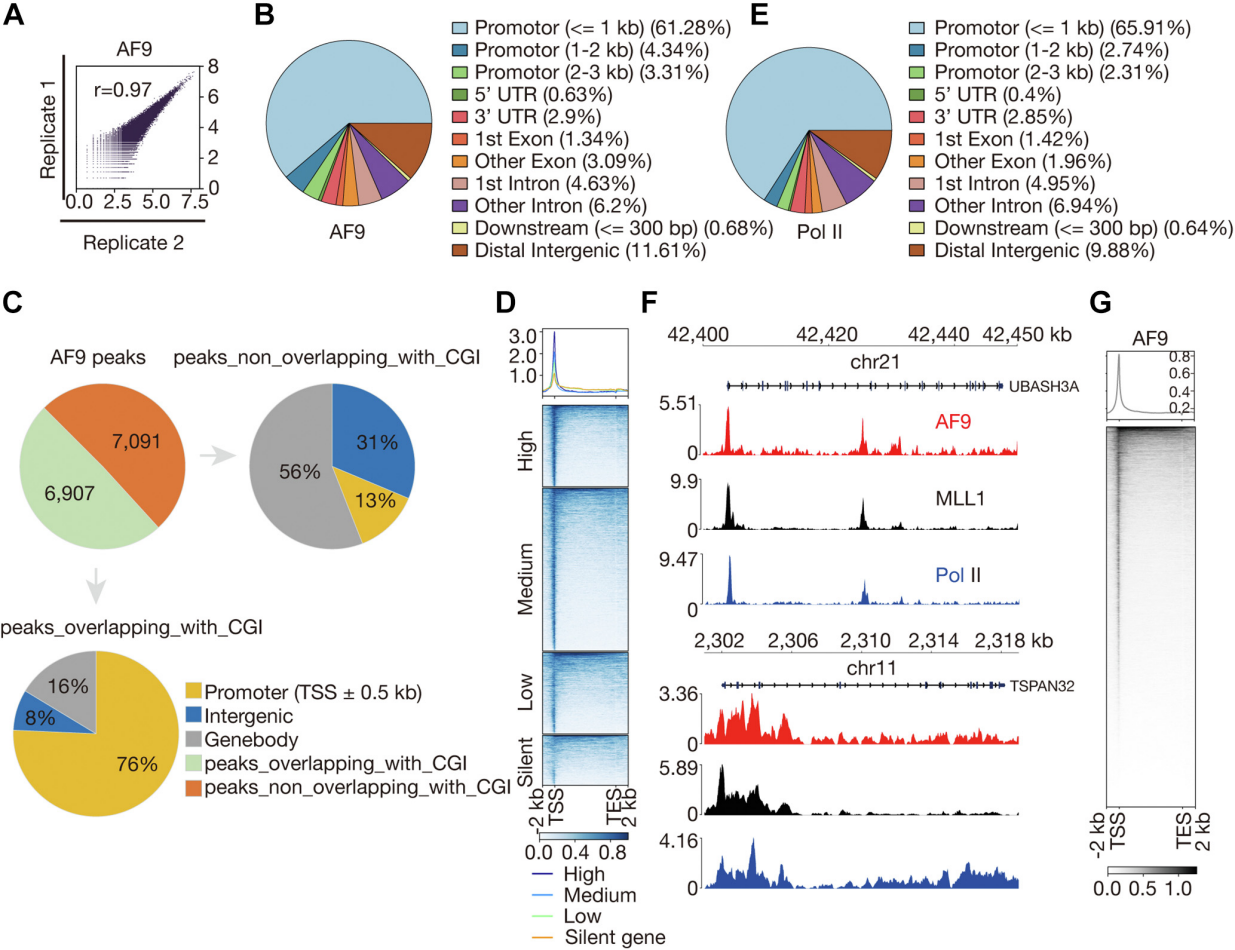
**AF9 mainly occupies TSSs in HEL cells.***A*, correlation plots for biological replicates of AF9 CUT&Tag experiments. Note that these CUT&Tag experiments actually had been performed in HEL cells transduced with a control shRNA, and that the plot has also been presented (reused) in Figure 4*J*, side by side with the related plot of knockdown cells, to make the results look complete, too. *B*, annotation of location of AF9 peaks in terms of genomic features. *C*, annotation of location of AF9 peaks in terms of CGI and TSS-proximal CGI. The *pie on the top left* shows number and percentage of AF9 peaks overlapping with CGI and nonoverlapping with CGI, the *pie at the bottom* shows genomic distribution of AF9 peaks that are overlapping with CGI, and the *pie on the top right* shows genomic distribution of AF9 peaks that are nonoverlapping with CGI. *D*, meta-gene profiles and heatmaps showing a positive correlation between AF9 occupancy near TSSs and mRNA levels of AF9 bound genes. Genes were sorted according to AF9 occupancy level detected by CUT&Tag. *High:* top 25% of the nonsilent genes (mean TPM ≥ 1); *medium:* nonsilent genes between top 25% and bottom 25%; *low:* bottom 25% of the nonsilent genes; and *silent:* genes with mean 0 < TPM < 1. *E*, annotation of location of Pol II peaks in terms of genomic features. *F*, normalized read distribution of AF9, MLL1, and Pol II within the *UBASH3A* and the *TSPAN32* loci. *G*, genome-wide meta-gene profiles and heatmaps of AF9 CUT&Tag experiments. CGI, CpG island; CUT&Tag, cleavage under target and tagmentation; HEL, human erythroleukemic; MLL1, mixed lineage leukemia 1; Pol II, polymerase II; TPM, transcripts per million; TSS, transcription start site.

A commercially available antibody AF9-N, raised against an N-terminus peptide of AF9, was used in CUT&Tag experiments of AF9 in HEL cells (Fig. 1*A*). Correlation analysis of related biological replicates indicates that the data are highly reproducible (Fig. 2*A*). With an FDR threshold of 0.01, we identified 14,019 peaks. To determine its chromatin occupancy pattern, we analyzed the overlap of peaks with known genomic features. 68.9%, 8.0%, 10.8%, and 11.6% of the peaks are overlapping with promoters, exons, introns, and distal intergenic regions, respectively (Fig. 2*B*). The association of a high percentage of AF9 peaks with promoters and the great overlap between promoters and CGIs also led us to analyze overlap between AF9 peaks and CGIs. 6907 (49.3%) of the peaks were found to overlap with CGIs, and 5367 (38%) of the peaks were found to overlap with TSS-proximal CGIs (Fig. 2*C*). The percentage of AF9 peaks associated with either CGI (49.3%) or TSS-proximal CGIs (38%) is lower than that of MLL1 (63% or 52%) (Fig. 1*D*), which indicates that AF9 occupancy is less dependent on CGIs and makes sense since AF9 contains no known CpG-binding motif. Together, these results suggest that AF9 mainly occupies TSSs.

To determine if there is any correlation between level of AF9 occupancy and mRNA level of genes, we performed comparative analysis and found a positive correlation between AF9 occupancy near TSSs and mRNA level of genes (Fig. 2*D*). Chromatin occupancy pattern of AF9 may be context dependent (26, 27). Comparative analysis of chromatin occupancy patterns of AF9 and Pol II revealed a great resemblance between them in HEL cells (Figs. 1, *F* and *G* and 2, *B*, *E*–*G*).

### MLL1 and AF9 coregulate a small number of genes

**Figure 3:**
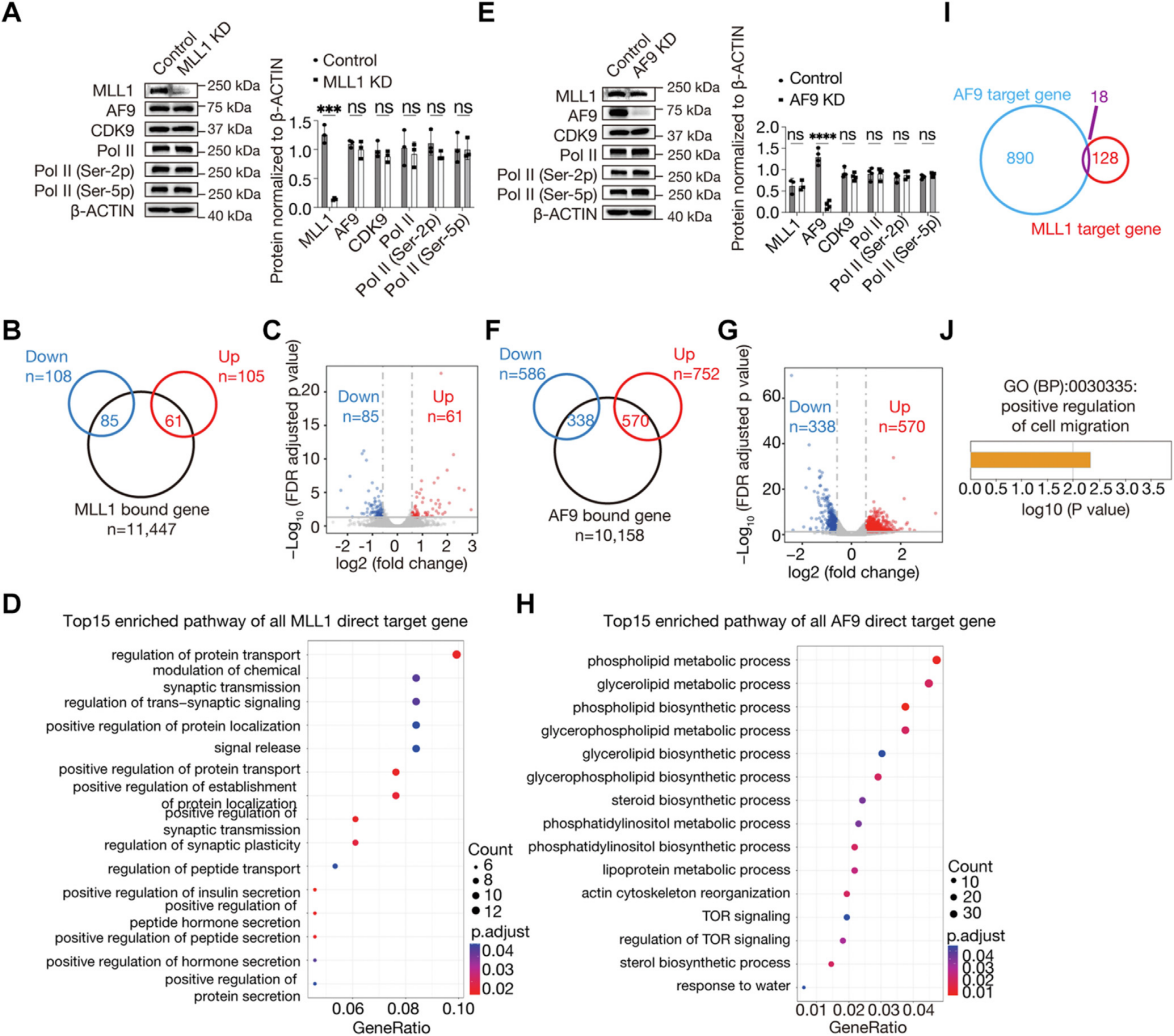
**MLL1 and AF9 coregulate a small number of genes in HEL cells.***A*, Western blot (WB) comparing cellular level of MLL1, AF9, Pol II, Pol II (Ser-2p), and Pol II (Ser-5p) in control and MLL1 KD cells. *Left:* pictures of the WB; *right:* WB results quantified by ImageJ (http://imagej.net/ij/). *B*, a *Venn diagram* showing overlaps between MLL1-bound genes identified by CUT&Tag and downregulated or upregulated genes identified by RNA-seq. *C*, a volcano plot showing gene expression changes under MLL1 deficiency. Significantly downregulated and upregulated genes are highlighted in *blue* and *red*, respectively. *D* and *H*, GO analysis results of direct target genes of MLL1 (*D*) and AF9 (*H*). *E*, WB comparing cellular level of MLL1, AF9, Pol II, Pol II (Ser-2p), and Pol II (Ser-5p) in control and AF9 KD cells. *Left:* pictures of the WB; *right:* WB results quantified by ImageJ. *F*, a *Venn diagram* showing overlaps between AF9-bound genes identified by CUT&Tag and downregulated or upregulated genes identified by RNA-seq. *G*, a volcano plot showing gene expression changes under AF9 deficiency. Significantly downregulated and upregulated genes are highlighted in *blue* and *red*, respectively. *I*, a *Venn diagram* showing an overlap between direct target genes of MLL1 and AF9. *J*, GO analysis result of direct target genes coregulated by AF9 and MLL1. Data shown are the mean ± SD of three independent experiments. Statistical significance was determined with a two-sided Student’s *t* test. NS: *p* ≥ 0.05, ∗*p* < 0.05, ∗∗*p* < 0.01, and ∗∗∗*p* < 0.001. CUT&Tag, cleavage under target and tagmentation; GO, gene ontology; KD, knockdown; MLL1, mixed lineage leukemia 1; Pol II, polymerase II; Ser-5p, serine 5 phosphorylated; Ser-2p, serine 2 phosphorylated.

To identify genes whose expressions are regulated by MLL1 in HEL cells, we initially planned to generate an HEL-based cell line with acute MLL1 degradation (36) through CRISPR-Cas9–mediated gene knockin. However, HEL cells seeded into wells of 96-well plates by serial dilution cannot form colonies, so that it is impossible to obtain the desired clones. We chose to use RNAi to knock MLL1 down afterward. The knockdown (KD) using one shRNA (MLL1 shRNA #1) was found to be efficient, and MLL1 deficiency had no effect on the cellular level of AF9, CDK9, total Pol II, CTD serine 5 phosphorylated (Ser-5p) Pol II, and CTD serine 2 phosphorylated (Ser-2p) Pol II (Fig. 3*A*). We subsequently compared mRNA in control and KD cells by RNA-seq. The numbers of significantly downregulated and upregulated genes were 108 and 105, respectively, in MLL1 KD cells relative to control cells with fold change ≥1.5 and FDR-adjusted *p* value <0.05 (Fig. 3*B*). Comparative analysis of MLL1 CUT&Tag data and the RNA-seq data identified 146 direct target genes of MLL1, defined as those with MLL1 occupancy from 2 kb upstream of their TSSs to 0.3 kb downstream of their transcription end sites (TESs) and showing significant expression change under MLL1 deficiency, and among them, 85 and 61 were downregulated and upregulated, respectively (Fig. 3, *B* and *C*). Moreover, gene ontology (GO) analysis of the direct target genes revealed enrichment of over one dozen terms, including regulation of protein transport, modulation of chemical synaptic transmission, positive regulation of protein transport, etc (Fig. 3*D*).

Similarly, to identify genes that are regulated by AF9 in HEL cells, we also knocked it down by a shRNA (AF9 shRNA #1). The KD was found to be efficient, and AF9 deficiency had no effects on the cellular level of MLL1, CDK9, Pol II, Pol II (Ser-5p), and Pol II (Ser-2p) (Fig. 3*E*). We subsequently compared mRNA in control and KD cells by RNA-seq. The numbers of significantly downregulated and upregulated genes were 586 and 752, respectively, in AF9 KD cells relative to control cells with fold change ≥1.5 and FDR-adjusted *p* value <0.05 (Fig. 3*F*). Comparative analysis of AF9 CUT&Tag data and the RNA-seq data identified 908 direct target genes, and among them, 338 and 570 were downregulated and upregulated, respectively (Fig. 3, *F* and *G*). Moreover, GO analysis of the direct target genes revealed enrichment of over one dozen terms (Fig. 3*H*). Furthermore, comparative analysis of direct target genes of MLL1 and direct target genes of AF9 identified 18 common direct targets genes (Fig. 3*I*); GO analyses revealed enrichment of only one term (Fig. 3*J*).

### MLL1 does not regulate the chromatin occupancy of the SEC

**Figure 4:**
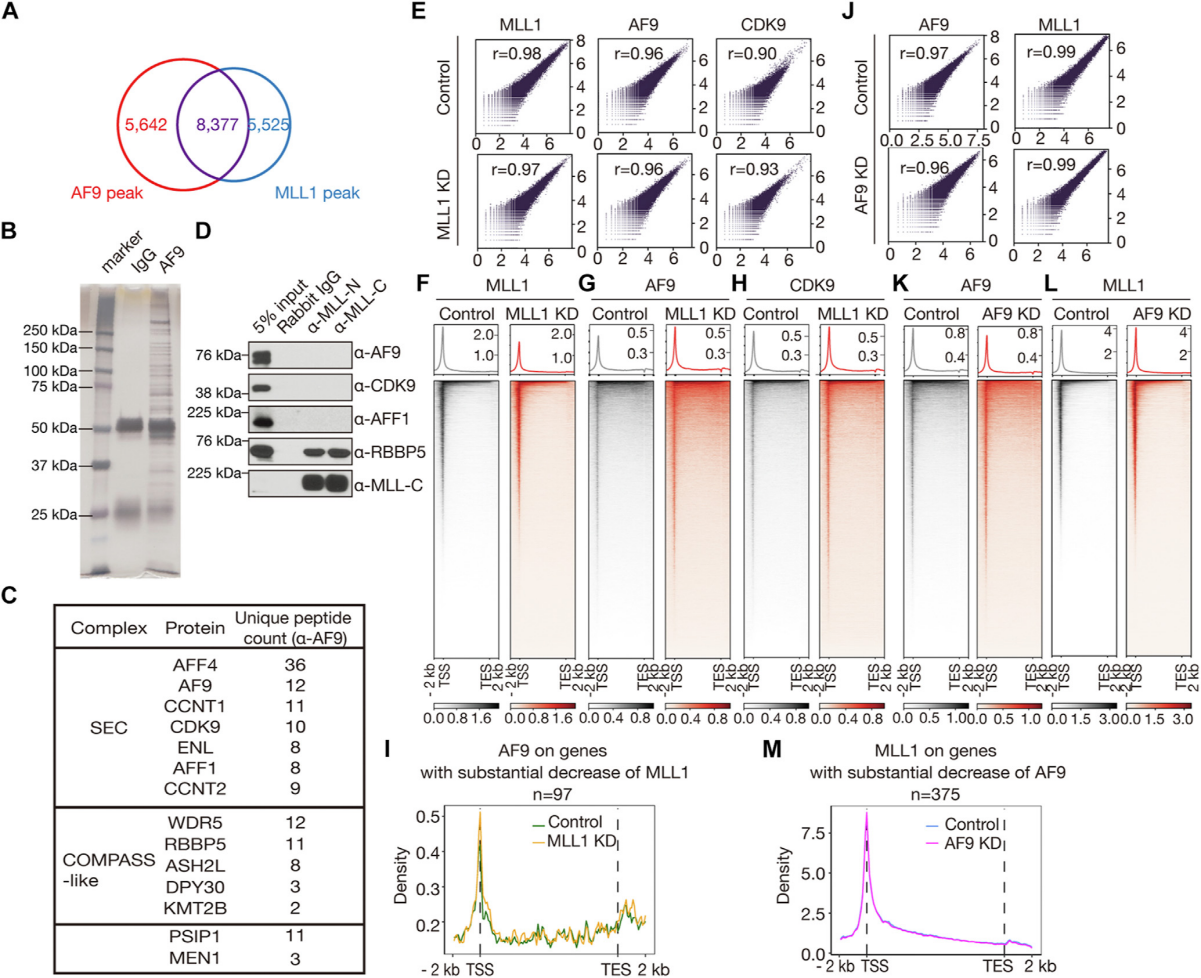
**Chromatin occupancy of MLL1 and AF9 are independent of each other in HEL cells.***A*, a *Venn diagram* showing an overlap between MLL1 and AF9 peaks. *B*, proteins immunoprecipitated by rabbit IgG (*lane 2*) and the AF9-N antibody (*lane 3*) separated on an SDS-PAGE gel. *C*, a table presenting a fraction of AF9-associated proteins. *D*, confirmation of some of the hits of the mass spectrometric analysis by regular Co-IP. *E*, correlation plots for biological replicates of MLL1 (*left*), AF9 (*middle*), and CDK9 (*right*) CUT&Tag experiments in control and MLL1 KD cells. *F*–*H*, genome-wide meta-gene profiles and heatmaps of MLL1 (*F*), AF9 (*G*), and CDK9 (*H*) CUT&Tag experiments in control and MLL1 KD cells. *I*, meta-gene profiles of AF9 occupancy on 97 genes exhibiting over 50% reduction of MLL1 occupancy in KD cells relative to that in control cells. *J*, correlation plots for biological replicates of AF9 (*left*) and MLL1 (*right*) CUT&Tag experiments in control and AF9 KD cells. *K* and *L*, genome-wide meta-gene profiles and heatmaps of AF9 (*K*) and MLL1 (*L*) CUT&Tag experiments in control and AF9 KD cells. *M*, meta-gene profiles of MLL1 occupancy on 375 genes exhibiting over 50% reduction of AF9 occupancy in KD cells relative to that in control cells. Co-IP, coimmunoprecipitation; CUT&Tag, cleavage under target and tagmentation; HEL, human erythroleukemic; KD, knockdown; MLL1, mixed lineage leukemia 1.

The great overlap between either MLL1 or AF9 peaks and TSSs led us to calculate overlap between peaks of MLL1 and AF9, and a great overlap was found. There are 8377 sites co-occupied by both factors, accounting for ∼60% of the MLL1 peaks and ∼60% of the AF9 peaks (Fig. 4*A*). However, it does not mean that they directly interact with each other in transcriptional regulation. To assess whether they directly interact with each other in HEL cells, we performed large-scale coimmunoprecipitation experiments using nuclear extract of HEL cells and the AF9-N antibody against the N terminus of AF9 (Fig. 1*A*) and analyzed immunoprecipitated proteins by mass spectrometry (Fig. 4*B*). Besides, AF9 and other subunits of the SEC, we also identified common subunits of the COMPASS-like complexes (WDR5, ASH2L, RBBP5, and DPY30) (11), KMT2B, menin, and lens epithelium derived growth factor, but no MLL1 (Fig. 4*C*). To confirm the results, we performed coimmunoprecipitation experiments individually using the MLL (468) antibody and an MLL1 antibody, MLL (456) (31), raised against an MLL-N fragment downstream of the break points (Fig. 1*A*), and found that AF9 and other SEC subunits were undetectable among proteins immunoprecipitated by the antibodies (Fig. 4*D*). Together, these results suggest that the probability that MLL1 and the SEC directly interact with each other in HEL cells is low, consistent with previous results (37).

To determine if MLL1 regulates chromatin occupancy of AF9, we performed CUT&Tag experiments for MLL1 and AF9 in control and MLL1 KD cells. Correlation analyses of related biological replicates indicate that the data are highly reproducible (Fig. 4*E*). We found that MLL1 KD greatly decreased chromatin occupancy of itself but minimally affected that of AF9 (Fig. 4, *F* and *G*). To determine whether MLL1 deficiency affects chromatin occupancy of other SEC subunits, we performed CUT&Tag for CDK9 in control and MLL1 KD cells and found that CDK9 occupancy was barely affected (Fig. 4, *E* and *H*). The numbers of MLL1-bound genes exhibiting significantly decreased and increased MLL1 occupancy near TSSs were 5188 and 6, respectively, and the corresponding numbers near TESs were 0 and 0, respectively (Fig. S1*A*). The numbers of MLL1-bound genes exhibiting significantly decreased and increased AF9 occupancy near TSSs were 0 and 2, respectively, and the corresponding numbers near TESs were 0 and 1, respectively (Fig. S1*B*). The numbers of MLL1-bound genes exhibiting significantly decreased and increased CDK9 occupancy near TSSs were 0 and 0, respectively, and the corresponding numbers near TESs were 0 and 0, respectively (Fig. S1*C*). To further examine if MLL1 deficiency affects AF9 occupancy, we focused on genes with over 50% MLL1 occupancy loss after MLL1 KD. There were 97 genes with over 50% reduction of MLL1 occupancy; MLL1 deficiency minimally affected AF9 occupancy on those genes (Fig. 4*I*), and the number of genes with significant AF9 occupancy changes was 0. These data suggest that in non-MLLr cells, MLL1 does not regulate recruitment of the SEC to chromatin.

To rule out the off-target effect of MLL1 shRNA #1, we knocked MLL1 down using another shRNA (MLL1 shRNA#2). The KD was found to be efficient, and its effects on cellular level of MLL1, AF9, Pol II, Pol II (Ser-2p), and Pol II (Ser-5p) were the same as those of MLL1 KD by shRNA#1 (Fig. S2*A*). Correlation analyses of related biological replicates indicate that the data are highly reproducible (Fig. S2*B*). We also found that although MLL1 KD by shRNA#2 reduced global occupancy of itself, MLL1 deficiency minimally affected AF9 occupancy (Fig. S2, *C* and *D*), consistent with the findings using shRNA #1.

### AF9 does not regulate the chromatin occupancy of MLL1

Conversely, to determine if AF9 regulates chromatin occupancy of MLL1, we performed CUT&Tag experiments for AF9 and MLL1 in control and AF9 KD cells. Correlation analyses of related biological replicates indicate that the data are highly reproducible (Fig. 4*J*). We found that AF9 KD greatly decreased chromatin occupancy of itself (Fig. 4*K*) but barely had any effect on MLL1 occupancy (Fig. 4*L*). The numbers of AF9-bound genes exhibiting significantly decreased and increased AF9 occupancy near TSSs were 927 and 10, respectively, and the corresponding numbers near TESs were 444 and 6, respectively (Fig. S1*D*). The numbers of AF9-bound genes exhibiting significantly decreased and increased MLL1 occupancy near TSSs were 1 and 1, respectively, and the corresponding numbers near TESs were 0 and 1, respectively (Fig. S1*E*). To further examine if AF9 deficiency affects MLL1 occupancy, we focused on genes with over 50% AF9 occupancy loss after AF9 KD. There were 375 genes with over 50% reduction of AF9 occupancy; AF9 deficiency had no effect on MLL1 occupancy on those genes (Fig. 4*M*), and the number of genes with significant MLL1 occupancy changes was 0.

### AF9 may regulate transcriptional initiation *via* facilitating TFIID recruitment in HEL cells

**Figure 5:**
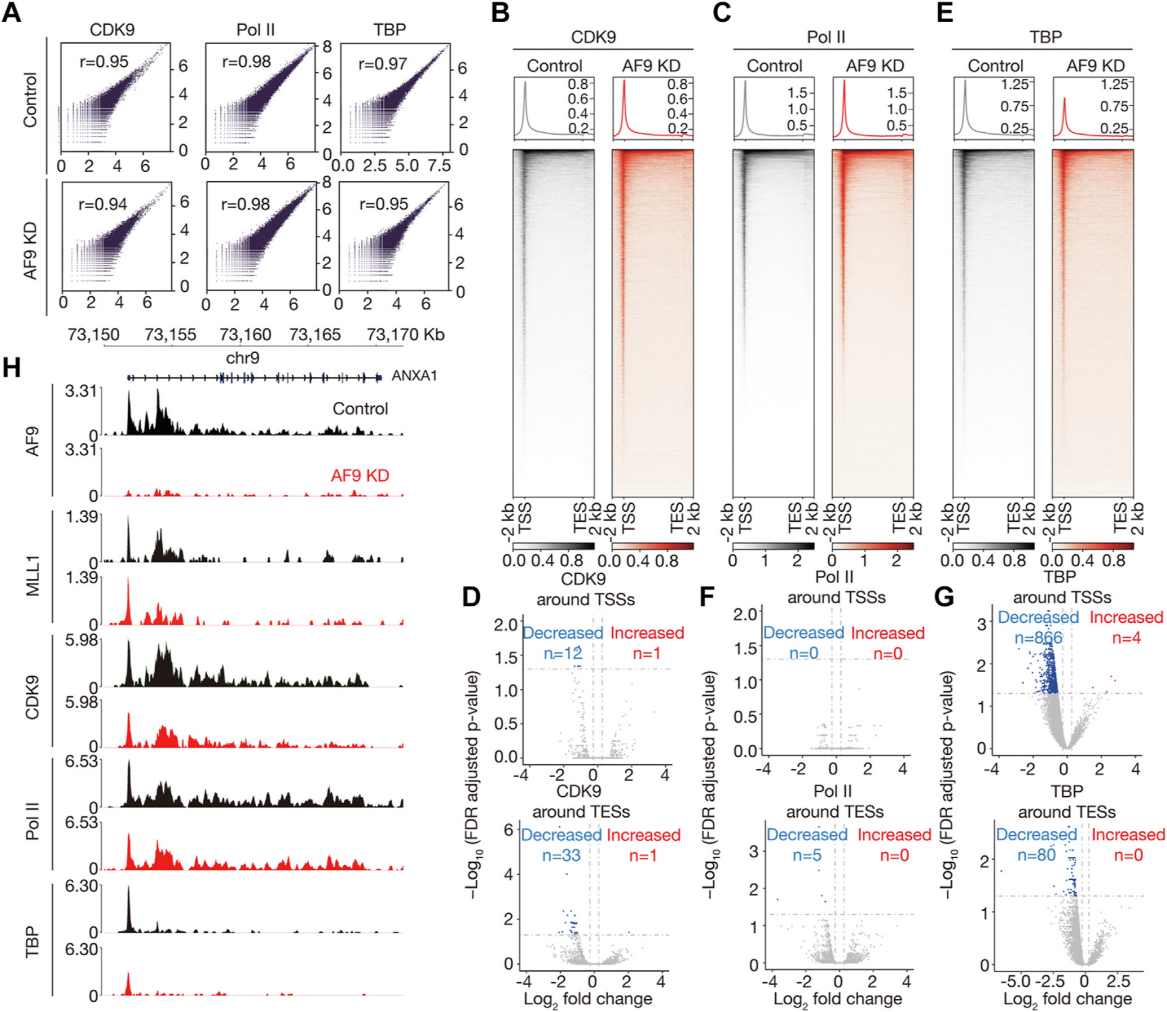
**AF9 may regulate transcription factor II D recruitment in HEL cells.***A*, correlation plots for biological replicates of CDK9 (*left*), Pol II (*middle*), and TBP (*right*) CUT&Tag experiments in control and AF9 KD cells. *B*, *C*, and *E*, genome-wide meta-gene profiles and heatmaps of CDK9 (*B*), Pol II (*C*), and TBP (*E*) CUT&Tag experiments in control and AF9 KD cells. *D*, *F*, and *G*, volcano plots showing occupancy changes of CDK9 (*D*), Pol II (*F*), and TBP (*G*) on AF9-bound genes in AF9 KD cells relative to control cells. *H*, normalized read distribution of AF9, MLL1, CDK9, Pol II, and TBP within the *ANXA1* locus in control and AF9 KD cells. CUT&Tag, cleavage under target and tagmentation; HEL, human erythroleukemic; KD, knockdown; MLL1, mixed lineage leukemia 1; Pol II, polymerase II.

The P-TEFb subunit of the SEC is a critical regulator of pause release, and the rest of the subunits are believed to affect pause release through P-TEFb (13). The global effect of AF9 loss on chromatin occupancy of P-TEFb, a heterodimer of CDK9 and cyclin T1, was undetermined. To this end, we performed CUT&Tag experiments of CDK9 in control and AF9 KD cells. Correlation analyses of related biological replicates indicate that the data are highly reproducible (Fig. 5*A*). We found that AF9 deficiency decreased CDK9 occupancy on a small number of genes (Fig. 5*B*). The numbers of AF9-bound genes exhibiting significantly decreased and increased CDK9 occupancy near TSSs were 12 and 1, respectively, and the corresponding numbers near TESs were 33 and 1, respectively (Fig. 5, *D* and *H*). Together, these results suggest that AF9 may play an accessory role in P-TEFb recruitment. The small effect on P-TEFb recruitment may also be due to the functional redundancy between AF9 and ENL.

To determine the effect of AF9 deficiency on Pol II occupancy, we performed CUT&Tag experiments for it in control and AF9 KD cells (Fig. 5*A*). We found that AF9 deficiency minimally affected global Pol II occupancy (Fig. 5*C*). The numbers of AF9-bound genes exhibiting significantly decreased and increased Pol II occupancy near TSSs were 0 and 0, respectively, and the corresponding numbers near TESs were 5 and 0, respectively (Fig. 5, *F* and *H*). Pausing index (PI), also known as traveling ratio, is defined as the ratio of promoter reads density to gene body reads density measured by genomic assays, including ChIP-seq, precision run-on sequencing, CUT&Tag, *etc.*, and has been used to measure the level of promoter-proximal pause of Pol II. To determine the effect of AF9 deficiency on pause of Pol II, we calculated PI for AF9-bound genes in control and AF9 KD cells, respectively, using the CUT&Tag data, and found that AF9 deficiency had little effect on pause of Pol II (Fig. S3*A*). The results were confirmed by precision run-on sequencing, which measures genomic distribution of engaged Pol II (Fig. S3*B*). Genes expressed in control cells were also divided into three groups, that is, highly paused (PI ≥ 3), moderately paused (1.5 ≤ PI < 3), and nonpaused (PI < 1.5), as previously described (38), and PI of genes in control and AF9 KD cells was compared group wisely. We found that only a subset of highly paused genes showed decrease of PI (Fig. S3*C*), suggesting decreased pause or initiation of Pol II.

We previously found that DOT1L or ENL KO in K562 cells decreases chromatin occupancy of TBP, one of the subunits of initiation factor, TFIID, and that the DOT1L complex may regulate transcriptional initiation in K562 cells (15). To evaluate if AF9 deficiency also affects initiation, we performed CUT&Tag for TBP in control and AF9 KD HEL cells (Fig. 5*A*). We found that AF9 deficiency decreased global TBP occupancy (Fig. 5*E*). The numbers of AF9-bound genes exhibiting significantly decreased and increased TBP occupancy near TSSs were 866 and 4, respectively, and the corresponding numbers near TESs were 80 and 0, respectively (Fig. 5, *G* and *H*). Together, these results suggest that AF9 regulates transcriptional initiation *via* facilitating TFIID recruitment in HEL cells. To assess whether decreased TBP occupancy would lead to decrease of mRNA level of genes, we performed comparative analysis of the RNA-seq data and the TBP CUT&Tag data. Among the 998 genes with decreased TBP occupancy upon AF9 KD (fold change ≥ 1.1), 53 were downregulated, 35 were upregulated, and 910 exhibited no expression change. With respect to why significantly reduced TBP occupancy on hundreds of genes (Fig. 5, *E* and *G*) upon AF9 KD only affected mRNA level of a much smaller number of genes in HEL cells, one explanation may be the compensatory enhancement of steps downstream of TFIID recruitment, and the other explanation is that the reductions of TBP occupancy were not great enough to cause significant expression changes.

To rule out the off-target effect of AF9 shRNA#1, we knocked AF9 down using another shRNA (AF9 shRNA#2). The KD was found to be efficient, and its effects on cellular level of MLL1, AF9, Pol II, Pol II (Ser-2p), and Pol II (Ser-5p) were the same as those of AF9 KD by shRNA#1 (Fig. S4*A*). Correlation analyses of related biological replicates indicate that the data are highly reproducible (Fig. S4*B*). We also found that AF9 KD by shRNA#2 reduced global occupancy of itself and TBP while had almost no effect on that of MLL1 (Fig. S4, *C*–*E*), consistent with the findings using shRNA #1.

### Chromatin occupancy pattern of MLL-AF9 is identical to that of MLL1

Several previous studies suggested differences between MLL1 and MLL-FPs with respect to location (23) and size (22, 28, 29) of occupancy sites, which are considered signatures of major target genes of MLL-FPs. However, chromatin occupancy patterns of MLL1 and major MLL-FPs have never been carefully compared in the same human MLL cells before. The major obstacle of comparing chromatin occupancy of MLL1 and MLL-AF9 in MLL cells is that there is no MLL-AF9–specific antibody available. Antibodies recognizing its MLL fragment would also recognize WT MLL1, and antibodies recognizing its AF9 fragment would also recognize WT AF9. Considering that AF9 mainly functions in hematopoietic stem or progenitor cells (30) and that its expression in most of the hematopoietic cell lines, including MLLr cell lines, are so low (39) that its loss may be of little consequence, we therefore chose to generate an AF9 KO cell line based on an AML cell line carrying one copy of (9;11) translocation for comparison of chromatin occupancy of MLL1 and MLL-AF9. MOLM-13 has been chosen over THP1 for its better colony-forming capability in wells of 96-well plates.

**Figure 6:**
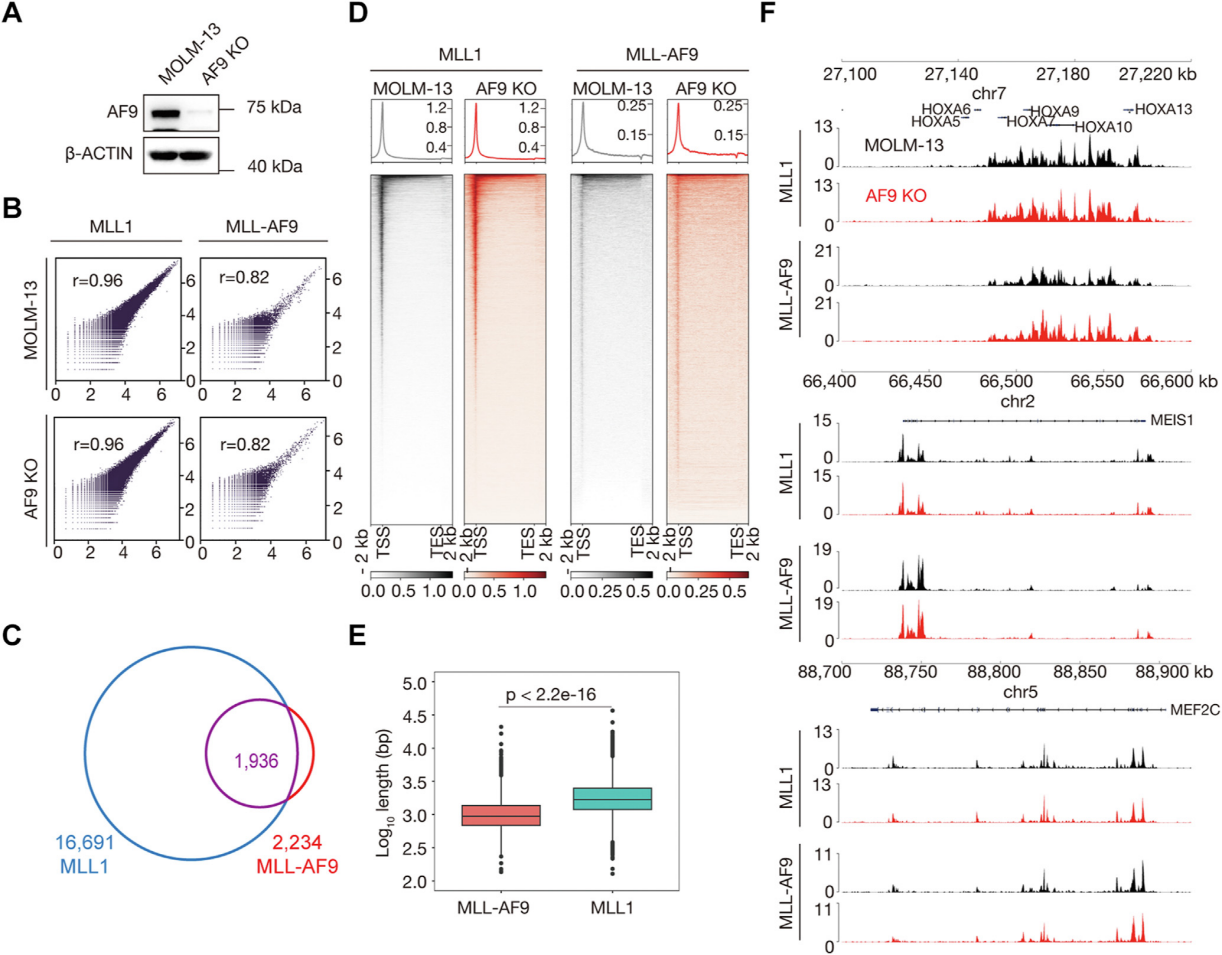
**Chromatin occupancy patterns of MLL1 and MLL-AF9 are identical in MOLM-13 cells.***A*, Western blot (WB) comparing cellular level of AF9 in MOLM-13 (control) and MOLM-13-AF9 KO cells. *B*, correlation plots for biological replicates of MLL1 (*left*) and MLL-AF9 (*right*) CUT&Tag experiments. *C*, a *Venn diagram* showing an overlap between peaks of MLL1 and MLL-AF9. *D*, genome-wide meta-gene profiles and heatmaps of MLL1 (*left*) and MLL-AF9 (*right*) CUT&Tag experiments. *E*, box plots comparing peak length (width) of MLL-AF9 and MLL1 in MOML13-AF9 KO cells. *F*, normalized read distribution of MLL1 and MLL-AF9 within the 5′ *HOXA*, *MEIS1*, and *MEF2C* loci. CUT&Tag, cleavage under target and tagmentation; MLL1, mixed lineage leukemia 1.

After obtaining the MOLM-13-AF9 KO cell line (Fig. 6*A*), we performed CUT&Tag for MLL1 [using MLL (456) antibody] and MLL-AF9 (using AF9-C antibody) in control and KO cells. Correlation analyses of related biological replicates indicate that the data are highly reproducible (Fig. 6*B*). With an FDR threshold of 0.01, we identified 16,691 and 2234 peaks for MLL1 and MLL-AF9, respectively, and the majority of the MLL-AF9 peaks (87%) are co-occupied by MLL1 (Fig. 6*C*). With respect to why peak number of MLL-AF9 is much smaller than that of MLL1, possible explanations include (1) the existence of unknown mechanisms in limiting chromatin occupancy of MLL-AF9 in MOLM-13-AF9 KO cells, and (2) the AF9-C antibody used for MLL-AF9 CUT&Tag is not as efficient as the MLL (456) antibody used for MLL1 CUT&Tag, as seen from the gel pictures of CUT&Tag (Fig. S5, *A* and *B*). Importantly, peaks of MLL-AF9 are not wider than those of MLL1, and there is no difference between their chromatin occupancy patterns in MOLM-13-AF9 KO cells (Fig. 6, *D*–*F*), contrary to what other people have found (22, 28, 29). The reason why MLL1 peaks are slightly wider than MLL-AF9 peaks may be the higher quality of the MLL1 CUT&Tag data.

### MLL-AF9 is also capable of regulating transcriptional initiation

To determine if MLL-AF9 is also capable of regulating transcriptional initiation, we had tried to generate an MLL-AF9-auxin-inducible degron (AID) cell line based on the MOLM-13-AF9 KO cell line. Unfortunately, we were unable to find an efficient way to electroporate plasmids into MOLM-13-AF9 KO cells. We had also tried to KD MLL-AF9 in MOLM-13-AF9 KO cells by lentiviral RNAi, but two high efficiency AF9 shRNAs in HEL cells were found to be inefficient in MOLM-13-AF9 KO cells (Fig. S6, *A* and *B*). Therefore, we chose a murine AML cell line, iMA9, which expresses C-terminus FLAG-tagged MLL-AF9 under the control of a Tet-off promoter (40).

**Figure 7:**
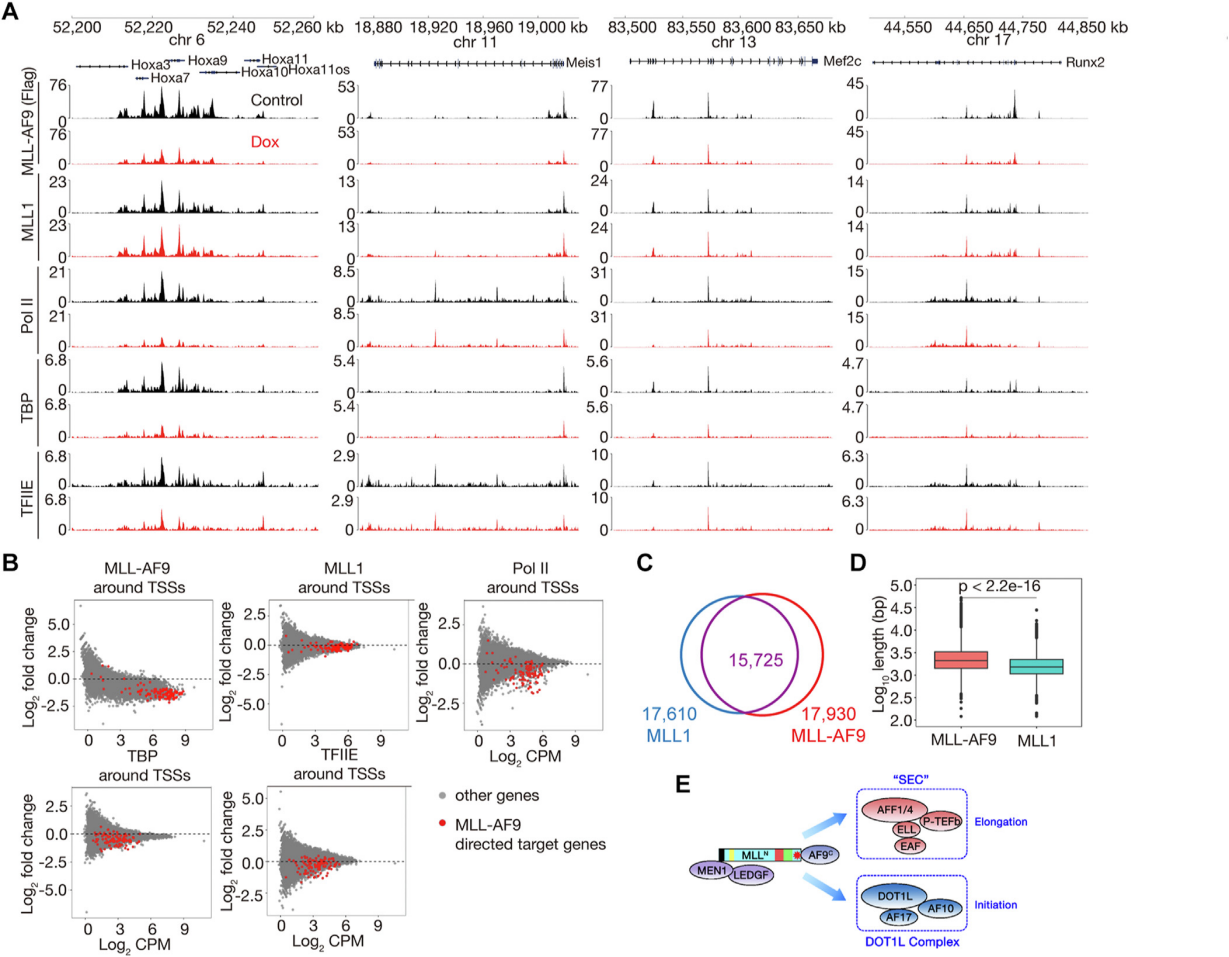
**MLL-AF9 may be capable of regulating transcriptional initiation.***A*, normalized read distribution of MLL-AF9, MLL1, Pol II, TBP, and TFIIE within the 5′ *Hoxa*, *Meis1*, *Mef2c*, and *Runx2* loci. *B*, MA plots showing occupancy changes of MLL-AF9, MLL1, Pol II, TBP, and TFIIE upon MLL-AF9 downregulation by doxycycline treatment. One hundred thirty-nine previously characterized target genes of MLL-AF9 are highlighted in *red*. *C*, a *Venn diagram* showing an overlap between peaks of MLL1 and MLL-AF9. *D*, box plots comparing peak length (width) of MLL-AF9 and MLL1 in iMA9 cells. *E*, model for the function MLL-AF9 in transcription. TSS-proximal CGI-bound MLL-AF9 facilitates transcriptional initiation and elongation by forming MLL-AF9–DOT1L complex and MLL-AF9-SEC, respectively. CGI, CpG island; DOT1L, disruptor of telomeric silencing 1-like; MLL1, mixed lineage leukemia 1; Pol II, polymerase II; SEC, super elongation complex; TSS, transcription start site.

We performed CUT&Tag experiments for MLL-AF9 (using FLAG M2 antibody), MLL1 [using MLL (456) antibody], Pol II, TBP, and TFIIE in untreated and doxycycline-treated iMA9 cells. Correlation analyses of related biological replicates indicate that the data are highly reproducible (Fig. S7*A*). We found that globally decreased MLL-AF9 occupancy had no global effect on occupancy of MLL1, Pol II, TBP, or TFIIE (Fig. S7*B*). However, most of the 139 previously reported MLL-AF9 target genes (41), including *Hoxa9*, *Meis1*, *Mef2c*, *Runx2*, *etc.*, exhibited decreased occupancy of Pol II, TBP, and TFIIE but unaffected that of MLL1 (Fig. 7, *A* and *B*), suggesting that MLL-AF9 is capable of regulating transcriptional initiation. With respect to why unlikely AF9 KD, MLL-AF9 downregulation did not decrease global occupancy of TBP, one explanation is that the AF9 part of MLL-AF9 only retains one of the two poly-serine stretches of AF9 (42).

By comparative analyses, we found that there are 15,725 peaks co-occupied by MLL1 and MLL-AF9, accounting for 89.3% of the MLL1 peaks and 87.7% of the MLL-AF9 peaks (Fig. 7*C*). MLL-AF9 peaks are slightly wider than MLL1 peaks in iMA9 cells (Fig. 7*D*), which is likely due to higher quality of the MLL-AF9 CUT&Tag data resulting from high-level expression of FLAG-tagged MLL-AF9 for immortalization of iMA9 and high efficiency of the FLAG M2 antibody used for MLL-AF9 CUT&Tag experiments.

## Discussion

It is believed that major MLL-FPs aberrantly maintain gene activation through simulating elongation (5). Previous studies also suggested differences between MLL1 and MLL-FPs with respect to location (23) and size (22, 28, 29) of occupancy sites, which are considered signatures of major target genes of MLL-FPs. In this study, we investigated not only the relationship between MLL1 and AF9, the most frequent fusion partner of MLL1 in AML, and the role of AF9 in transcriptional regulation in the non-MLLr HEL cells, but also the chromatin occupancy patterns of MLL1 and MLL-AF9, and the role of MLL-AF9 in transcriptional regulation in MLL cells. We found that there is no difference between MLL1 and MLL-AF9 with respect to location and size of occupancy sites and that MLL-AF9 may be capable of regulating transcriptional initiation in addition to elongation (Fig. 7*E*).

### The SEC may play an important role in initiation besides elongation

AF9 and its paralog, ENL, are major fusion partners of MLL1 in MLL. They can be integrated into the SEC and the DOT1L complex in a mutually exclusive manner. The P-TEFb subunit of the SEC is a critical regulator of pause release of Pol II (13). H3K79 dimethylation, catalyzed by DOT1L, has been found to be associated with the 5′ regions of active genes (43), leading to the view of DOT1L as an elongation factor. Most of the noncatalytic subunits of the SEC or the DOT1L complex have been found to be able to affect the recruitment or the activity of the corresponding catalytic subunit, so they are considered regulators of transcriptional elongation. It has been shown before that two poly-serine stretches of AF9 are capable of directly interacting with TFIID (42); we previously found that DOT1L or ENL knockout decreased TFIID recruitment and the DOT1L complex may regulate transcriptional initiation in human K562 cells (15). In this study, we found that AF9 KD significantly decreased TBP occupancy on hundreds of genes while affected CDK9 occupancy on a much smaller number of genes in human HEL cells, suggesting a role of AF9 in initiation besides elongation. These results together suggest that the SEC may also play an important role in initiation through AF9 or ENL besides a well-established critical role in elongation through P-TEFb.

With respect to why significantly reduced TBP occupancy on hundreds of genes (Fig. 5, *E* and *G*) upon AF9 KD only affected Pol II occupancy on a much smaller number of genes (Fig. 5, *C* and *F*) in HEL cells, one explanation may be the compensatory enhancement of steps downstream of TFIID recruitment, and another explanation may be the opposite effects of decreased initiation, which decreases Pol II occupancy, and decreased pause release caused by compromised P-TEFb function, which increases Pol II occupancy.

### MLL-AF9 may be capable of regulating transcriptional initiation and elongation

One of the earlier studies performed ChIP-seq experiments for MLL1 and AF4 (also known as AFF1) in two human cell lines, the SEM cell line, harboring a (4;11)(q21;q23) translocation, and the REH cell line, harboring no MLLr, respectively; target genes of MLL-AF4 were defined as those co-occupied by both MLL1 and AF4, and the authors found that regions occupied by MLL-AF4 (5–100 kb) are much larger in size than regions occupied by MLL1 or AF4 (1–3 kb) (22). Another earlier study performed MLL1 ChIP-chip in three human cell lines with *MLL1* translocations, ML-2, MV4;11 and THP1-expressing MLL-AF6, MLL-AF4, and MLL-AF9, respectively, and two human cell lines without MLLr, HL60, and U937; the authors found that MLL-FPs preferentially occupy a small proportion of the leukemic genome with high level and an extended distribution pattern (28). One recent study also performed ChIP-seq experiments for MLL1 and AF4 in SEM cells; the authors found that 3.4% of the target genes exhibit spreading occupancy of MLL-AF4 greater than 4 kb onto gene bodies, those genes are more dependent on MLL-AF4, and the spreading is specific for MLL-FPs, not MLL1 (29). Moreover, one study using murine AML cells expressing MLL-AF9 discovered that MLL1 binds to chromatin regions distinct from those of MLL-AF9 (23). Nonetheless, occupancy of MLL1 and MLL-FPs had never been carefully compared in the same human cells harboring MLLr.

The major obstacle of comparing chromatin occupancy of MLL1 and MLL-AF9 in MLL cells is that there is no MLL-AF9–specific antibody available. Taking advantage of the MOLM-13-AF9 KO cell line, the MLL (468) antibody and the AF9-C antibody, we found that chromatin occupancy patterns of MLL1 and MLL-AF9 are very likely to be identical, and there is no difference between them with respect to location and size of occupancy sites (Fig. 6, *D*–*F*). This conclusion is supported by CUT&Tag results of MLL1 and MLL-AF9 in iMA9 cells (Figs. S7*B* and 7, *A*, and *D*). These results suggest that even if MLL-AF9 is capable of aberrantly stimulating elongation, it is unlikely through traveling with Pol II alone gene body *via* its AF9 part. Considering that MLL1 and AF9 do not directly interact with each other in non-MLLr cells, in our view, it is more likely that the fusion between them in MLLr cells creates an unbreakable shortcut for transcriptional initiation and elongation bypassing the need of proteins working between them in non-MLLr cells for gene activation (Fig. 7*E*). Future works are needed to determine if other major MLL-FPs, in particular MLL-AF4, are also capable of regulating transcriptional initiation.

## Limitation of this study

It is ideal to use an acute protein degradation system, most notably, the AID system, to determine the direct effects of the loss of a transcription or epigenetic factor. Obtaining cells harboring the AID system need genome editing and single-cell cloning. However, HEL cells seeded into wells of 96-well plates by serial dilution cannot form colonies, so that it is impossible to obtain the desired cells. Our conclusion that AF9 regulates TFIID recruitment was drawn from genomic experiments using control and AF9 KD cells, so the effect might not be immediate.

## Experimental procedures

### Cell culture

Human cells 293T were cultured in 90% Dulbecco's modified Eagle's medium + 10% fetal bovine serum + 2% Penicillin-Streptomycin + 2 mM L-Glutamine. Human cells MOLM-13 and HEL and murine cells iMA9 were cultured in 90% Roswell Park Memorial Institute-1640 medium + 10% fetal bovine serum + 2% Penicillin-Streptomycin + 2 mM L-Glutamine + 55 μM β-mercaptoethanol. For downregulating expression of MLL-AF9, doxycycline was added to iMA9 cells at a concentration of 0.5 μg/ml.

### Lentiviral RNAi

For lentivirus production and transduction, 60 to 90% confluent 293T cells in antibiotic-free medium were transfected on day 1 with the TRC control and a gene-specific lentiviral shRNA plasmid, respectively, together with packaging plasmids psPAX2 and pMD2.G. On day 2 in the morning, medium-containing transfection reagent was replaced with fresh medium-containing 2% Penicillin-Streptomycin (Sigma-Aldrich, cat. no. P0781). On day 3 in the afternoon, HEL cells were resuspended in virus-containing medium and spun at 2000 rpm at 20 °C for 1 h for infection. After infection, virus-containing medium was removed; the cells were resuspended in fresh medium and cultured overnight. On day 4 in the morning, the cells were washed twice with PBS and resuspended in fresh medium. On day 4 in the afternoon, the cells were transduced with the same viruses again. On day 5 in the morning, the cells were washed twice with PBS, resuspended in fresh medium. On day 6 in the morning, puromycin was added to a final concentration of 2 μg/ml. The cells were cultured for additional 72 h before being harvested for quantitative reverse transcription polymerase chain reaction, Western blot and CUT&Tag. Mission shRNA clones used in this study are listed in Table S1. Antibodies used for Western blot experiments are listed in Table S2. Primers for real time PCR are listed in Table S3.

## Data availability

Next generation sequencing data have been submitted to Gene Expression Omnibus repository under accession number GSE231814. The mass spectrometry proteomics data have been deposited to the ProteomeXchange Consortium *via* the PRIDE (44) partner repository with the dataset identifier PXD042008.

## Supporting information

This article contains supporting information (45, 46, 47, 48, 49, 50, 51, 52, 53, 54, 55, 56, 57, 58).

## Conflict of interest

The authors declare that they have no conflicts of interest with the contents of this article.

## References

[1] Issa G.C., Ravandi F., DiNardo C.D., Jabbour E., Kantarjian H.M., Andreeff M. (2021). Therapeutic implications of menin inhibition in acute leukemias. Leukemia.

[2] Yagi H., Deguchi K., Aono A., Tani Y., Kishimoto T., Komori T. (1998). Growth disturbance in fetal liver hematopoiesis of Mll-mutant mice. Blood.

[3] Ernst P., Fisher J.K., Avery W., Wade S., Foy D., Korsmeyer S.J. (2004). Definitive hematopoiesis requires the mixed-lineage leukemia gene. Dev. Cell.

[4] Jude C.D., Climer L., Xu D., Artinger E., Fisher J.K., Ernst P. (2007). Unique and independent roles for MLL in adult hematopoietic stem cells and progenitors. Cell Stem Cell.

[5] Krivtsov A.V., Hoshii T., Armstrong S.A. (2017). Mixed-lineage leukemia fusions and chromatin in leukemia. Cold Spring Harb. Perspect. Med..

[6] Hsieh J.J., Cheng E.H., Korsmeyer S.J. (2003). Taspase1: a threonine aspartase required for cleavage of MLL and proper HOX gene expression. Cell.

[7] Rao R.C., Dou Y. (2015). Hijacked in cancer: the KMT2 (MLL) family of methyltransferases. Nat. Rev. Cancer.

[8] Issa G.C., Aldoss I., DiPersio J., Cuglievan B., Stone R., Arellano M. (2023). The menin inhibitor revumenib in KMT2A-rearranged or NPM1-mutant leukaemia. Nature.

[9] Wang X., Chen C.W., Armstrong S.A. (2016). The role of DOT1L in the maintenance of leukemia gene expression. Curr. Opin. Genet. Dev..

[10] Zhao D., Li Y., Xiong X., Chen Z., Li H. (2017). YEATS domain-A histone acylation reader in Health and disease. J. Mol. Biol..

[11] Smith E., Lin C., Shilatifard A. (2011). The super elongation complex (SEC) and MLL in development and disease. Genes Dev..

[12] Liu Z., Wu A., Wu Z., Wang T., Pan Y., Li B. (2022). TOX4 facilitates promoter-proximal pausing and C-terminal domain dephosphorylation of RNA polymerase II in human cells. Commun. Biol..

[13] Core L., Adelman K. (2019). Promoter-proximal pausing of RNA polymerase II: a nexus of gene regulation. Genes Dev..

[14] Luo Z., Lin C., Shilatifard A. (2012). The super elongation complex (SEC) family in transcriptional control. Nat. Rev. Mol. Cell Biol..

[15] Wu A., Zhi J., Tian T., Cihan A., Cevher M.A., Liu Z. (2021). DOT1L complex regulates transcriptional initiation in human erythroleukemic cells. Proc. Natl. Acad. Sci. U. S. A..

[16] Zeisig B.B., Milne T., García-Cuéllar M.P., Schreiner S., Martin M.E., Fuchs U. (2004). Hoxa9 and Meis1 are key targets for MLL-ENL-mediated cellular immortalization. Mol. Cell Biol..

[17] Ramos-Mejia V., Navarro-Montero O., Ayllón V., Bueno C., Romero T., Real P.J. (2014). HOXA9 promotes hematopoietic commitment of human embryonic stem cells. Blood.

[18] Wang H., Liu C., Liu X., Wang M., Wu D., Gao J. (2018). MEIS1 regulates hemogenic endothelial generation, megakaryopoiesis, and thrombopoiesis in human pluripotent stem cells by targeting TAL1 and FLI1. Stem Cell Rep..

[19] Sauvageau G., Lansdorp P.M., Eaves C.J., Hogge D.E., Dragowska W.H., Reid D.S. (1994). Differential expression of homeobox genes in functionally distinct CD34+ subpopulations of human bone marrow cells. Proc. Natl. Acad. Sci. U. S. A..

[20] Pineault N., Helgason C.D., Lawrence H.J., Humphries R.K. (2002). Differential expression of Hox, Meis1, and Pbx1 genes in primitive cells throughout murine hematopoietic ontogeny. Exp. Hematol..

[21] Ayton P.M., Cleary M.L. (2003). Transformation of myeloid progenitors by MLL oncoproteins is dependent on Hoxa7 and Hoxa9. Genes Dev..

[22] Guenther M.G., Lawton L.N., Rozovskaia T., Frampton G.M., Levine S.S., Volkert T.L. (2008). Aberrant chromatin at genes encoding stem cell regulators in human mixed-lineage leukemia. Genes Dev..

[23] Xu J., Li L., Xiong J., denDekker A., Ye A., Karatas H. (2016). MLL1 and MLL1 fusion proteins have distinct functions in regulating leukemic transcription program. Cell Discov..

[24] Terranova R., Agherbi H., Boned A., Meresse S., Djabali M. (2006). Histone and DNA methylation defects at Hox genes in mice expressing a SET domain-truncated form of Mll. Proc. Natl. Acad. Sci. U. S. A..

[25] Mishra B.P., Zaffuto K.M., Artinger E.L., Org T., Mikkola H.K.A., Cheng C. (2014). The histone methyltransferase activity of MLL1 is dispensable for hematopoiesis and leukemogenesis. Cell Rep..

[26] Lin C., Garrett A.S., De Kumar B., Smith E.R., Gogol M., Seidel C. (2011). Dynamic transcriptional events in embryonic stem cells mediated by the super elongation complex (SEC). Genes Dev..

[27] Yu M., Yang W., Ni T., Tang Z., Nakadai T., Zhu J., Roeder R.G. (2015). RNA polymerase II-associated factor 1 regulates the release and phosphorylation of paused RNA polymerase II. Science.

[28] Wang Q.F., Wu G., Mi S., He F., Wu J., Dong J. (2011). MLL fusion proteins preferentially regulate a subset of wild-type MLL target genes in the leukemic genome. Blood.

[29] Kerry J., Godfrey L., Repapi E., Tapia M., Blackledge N.P., Ma H. (2017). MLL-AF4 spreading identifies binding sites that are distinct from super-enhancers and that govern sensitivity to DOT1L inhibition in leukemia. Cell Rep..

[30] Calvanese V., Nguyen A.T., Bolan T.J., Vavilina A., Su T., Lee L.K. (2019). MLLT3 governs human haematopoietic stem-cell self-renewal and engraftment. Nature.

[31] Blobel G.A., Kadauke S., Wang E., Lau A.W., Zuber J., Chou M.M. (2009). A reconfigured pattern of MLL occupancy within mitotic chromatin promotes rapid transcriptional reactivation following mitotic exit. Mol. Cell.

[32] Ayton P.M., Chen E.H., Cleary M.L. (2004). Binding to nonmethylated CpG DNA is essential for target recognition, transactivation, and myeloid transformation by an MLL oncoprotein. Mol. Cell Biol..

[33] Deaton A.M., Bird A. (2011). CpG islands and the regulation of transcription. Genes Dev..

[34] Illingworth R.S., Bird A.P. (2009). CpG islands--'a rough guide'. FEBS Lett..

[35] Barski A., Cuddapah S., Cui K., Roh T.Y., Schones D.E., Wang Z. (2007). High-resolution profiling of histone methylations in the human genome. Cell.

[36] Yesbolatova A., Saito Y., Kitamoto N., Makino-Itou H., Ajima R., Nakano R. (2020). The auxin-inducible degron 2 technology provides sharp degradation control in yeast, mammalian cells, and mice. Nat. Commun..

[37] Thiel A.T., Blessington P., Zou T., Feather D., Wu X., Yan J. (2010). MLL-AF9-induced leukemogenesis requires coexpression of the wild-type Mll allele. Cancer Cell.

[38] Yu L., Zhang B., Deochand D., Sacta M.A., Coppo M., Shang Y. (2020). Negative elongation factor complex enables macrophage inflammatory responses by controlling anti-inflammatory gene expression. Nat. Commun..

[39] Prange K.H.M., Mandoli A., Kuznetsova T., Wang S.Y., Sotoca A.M., Marneth A.E. (2017). MLL-AF9 and MLL-AF4 oncofusion proteins bind a distinct enhancer repertoire and target the RUNX1 program in 11q23 acute myeloid leukemia. Oncogene.

[40] Zuber J., Rappaport A.R., Luo W., Wang E., Chen C., Vaseva A.V. (2011). An integrated approach to dissecting oncogene addiction implicates a Myb-coordinated self-renewal program as essential for leukemia maintenance. Genes Dev..

[41] Bernt K.M., Zhu N., Sinha A.U., Vempati S., Faber J., Krivtsov A.V. (2011). MLL-rearranged leukemia is dependent on aberrant H3K79 methylation by DOT1L. Cancer Cell.

[42] Yadav D., Ghosh K., Basu S., Roeder R.G., Biswas D. (2019). Multivalent role of human TFIID in recruiting elongation components at the promoter-proximal region for transcriptional control. Cell Rep..

[43] Steger D.J., Lefterova M.I., Ying L., Stonestrom A.J., Schupp M., Zhuo D. (2008). DOT1L/KMT4 recruitment and H3K79 methylation are ubiquitously coupled with gene transcription in mammalian cells. Mol. Cell Biol..

[44] Perez-Riverol Y., Bai J., Bandla C., García-Seisdedos D., Hewapathirana S., Kamatchinathan S. (2022). The PRIDE database resources in 2022: a hub for mass spectrometry-based proteomics evidences. Nucleic Acids Res..

[45] Rahl P.B., Lin C.Y., Seila A.C., Flynn R.A., McCuine S., Burge C.B. (2010). c-Myc regulates transcriptional pause release. Cell.

[46] Kwak H., Fuda N.J., Core L.J., Lis J.T. (2013). Precise maps of RNA polymerase reveal how promoters direct initiation and pausing. Science.

[47] Yu M., Mazor T., Huang H., Huang H.T., Kathrein K.L., Woo A.J. (2012). Direct recruitment of polycomb repressive complex 1 to chromatin by core binding transcription factor. Mol. Cell.

[48] Zhong S., Joung J.G., Zheng Y., Chen Y.R., Liu B., Shao Y. (2011). High-throughput illumina strand-specific RNA sequencing library preparation. Cold Spring Harb. Protoc..

[49] Chen S., Zhou Y., Chen Y., Gu J. (2018). fastp: an ultra-fast all-in-one FASTQ preprocessor. Bioinformatics.

[50] Kim D., Paggi J.M., Park C., Bennett C., Salzberg S.L. (2019). Graph-based genome alignment and genotyping with HISAT2 and HISAT-genotype. Nat. Biotechnol..

[51] Liao Y., Smyth G.K., Shi W. (2014). featureCounts: an efficient general purpose program for assigning sequence reads to genomic features. Bioinformatics.

[52] Love M.I., Huber W., Anders S. (2014). Moderated estimation of fold change and dispersion for RNA-seq data with DESeq2. Genome Biol..

[53] Wang T., Zhao R., Zhi J., Liu Z., Wu A., Yang Z. (2023). Tox4 regulates transcriptional elongation and reinitiation during murine T cell development. Commun. Biol..

[54] Langmead B., Salzberg S.L. (2012). Fast gapped-read alignment with Bowtie 2. Nat. Methods.

[55] Li H., Handsaker B., Wysoker A., Fennell T., Ruan J., Homer N. (2009). The sequence alignment/map format and SAMtools. Bioinformatics.

[56] Meers M.P., Tenenbaum D., Henikoff S. (2019). Peak calling by Sparse Enrichment Analysis for CUT&RUN chromatin profiling. Epigenetics Chromatin.

[57] Robinson M.D., McCarthy D.J., Smyth G.K. (2009). edgeR: a bioconductor package for differential expression analysis of digital gene expression data. Bioinformatics.

[58] Yu M., Riva L., Xie H., Schindler Y., Moran T.B., Cheng Y. (2009). Insight into GATA-1-mediated gene activation versus repression via genome-wide chromatin occupancy analysis. Mol. Cell.

