# Supplementary material for: MLL-AF9 regulates transcriptional initiation in mixed lineage leukemic cells
Source: J Biol Chem. 2024 Jul 11;300(8):107566. doi: 10.1016/j.jbc.2024.107566 (PMC11345648; doi:10.1016/j.jbc.2024.107566)
Supplement: Supporting information [file mmc1.docx]

**Supporting Information**

**Supplementary Methods**

*Design and clone MLL1 and AF9 shRNAs into pLKO.1*

One shRNA targeting sequence “TGCCAAGCACTGTCGAAATTA” of MLL1 (NM_001197104.2) and one shRNA targeting sequence “TTCAAAGCCTCACAAATTAAT” of AF9 (NM_004529.3) were designed through the public portal of The RNAi Consortium. Synthesized oligos after annealing were cloned into pLKO.1 through the *Age*I and the *Eco*RI cutting sites. pLKO.1 TRC control (SHC016) and the rest of the shRNA expressing plasmids used in this study were purchased from Sigma Aldrich.

*Generation of knockout cell line by CRISPR-Cas9*

Guide RNAs (gRNAs) were designed using the tool provided by Benchling. Oligos were annealed and cloned into lentiCRISPR v2 (Addgene, Plasmid #52961). Lentiviruses were prepared as previously described^47^. Single colonies obtained by serial dilution were expanded and subsequently characterized by Western blot. Oligos for gRNA cloning used in this study are listed in Supplementary Table 1.

*RNA extraction, reverse transcription, real-time PCR, RNA-seq and related analysis*

RNA was extracted from cells using RNeasy Plus Mini Kit (Qiagen, cat. no. 74134) and Quick-RNA MiniPrep Kit (Zymo Research, cat. no. R1054) by following the manufacturers’ protocols. For RT-PCR, single-strand cDNA was synthesized using iScript cDNA Synthesis Kit (Bio-Rad, cat. no. 1708891). For real-time PCR, β-Actin was used as the internal control. Relative abundance of transcript was calculated by the 2^ΔCt^ method. Libraries of strand-specific RNA-seq were constructed as previously described^48^. Raw reads were filtered using fastp^49^ (version 0.13.1, default parameters) and mapped to hg38 using HISAT2^50^ (version 2.1.0) with parameters “--rna-strandness RF –dta”. Read counts per gene were calculated in strand-specific manner using featureCounts^51^. Differential expression analysis was performed using DESeq2^52^, and genes with mean TPM ≥ 1, FDR < 0.05 and fold change ≥ 1.5 were identified as significantly differentially expressed.

*CUT&Tag and related analysis*

CUT&Tag experiments were performed as previously described with minor modifications^53^. Briefly, 100,000 human cells or 200,000 murine cells were used for each experiment. Cells were bound to Concanavalin A-coated beads without fixation and chromatin opening. After primary and secondary antibodies binding, pA-Tn5 transposome binding, and tagmentation, DNA was extracted and amplified by PCR. Antibodies used for CUT&Tag experiments are listed in Table S2.

Raw reads were filtered using fastp (version 0.13.1, default parameters) ^49^ and aligned to human genome hg38 using Bowtie2 (version 2.3.4.1) ^54^. Low-quality alignments were filtered out using SAMtools (version 0.1.19) ^55^ with command “samtools view -F 1804 -q 25”. MarkDuplicates tools in Picard was used to identify and remove PCR duplicates from the aligned reads. Peak calling was performed using SEACR (version 1.3) ^56^ with an FDR threshold 0.01 in stringent mode. Peaks were called initially from merged reads of two biological replicates, and among them, those cannot be called subsequently from either of the biological replicates were removed. The remaining peaks were defined as high confidence ones. A gene is considered as bound by one factor if any peak of this factor is found from 2 kb upstream to 0.3 kb downstream of this gene. EdgeR^57^ was used to analyze differential occupancy around TSSs (from 2 kb upstream to 2 kb downstream of TSSs) or TESs (from 2 kb upstream to 2 kb downstream of TESs), and genes with a BH-adjusted p value < 0.05 and fold change ≥ 1.2 (or ≥ 1.1 when specified) were identified as differentially bound. Antibodies for CUT&Tag are listed in Table S2.

With respect to MA plots, the log_2_fold-change and log_2_CPM of MLL-AF9, MLL1, Pol II, TBP and TFIIE occupancy on promoters (from 2 kb upstream to 2 kb downstream of TSSs) were calculated by edgeR (v3.24.3), respectively. With respect to box plots of peak length (width), the p-values were calculated by unpaired Wilconx test.

*PRO-seq and related analysis*

PRO-seq experiments were performed as previously described^39^, and the libraries were sequenced by Illumina HiSeq 2500. Raw reads were mapped to reference genome (hg38) using Bowtie2. To visualize engaged Pol II occupancies on genes by aggregation plots (average gene plots), genes with length greater than 500 bp and RPKM >1 (determined by RNA-seq) were selected; gene bodies were split into 300 windows, and regions 2 kb upstream of transcription start site (TSS) and 2 kb downstream of transcription end sites (TES) were split into 50 windows, respectively; reads density within each window was calculated separately for control and knockdown samples, and normalized to sequencing depth; normalized reads density values were used to draw the aggregation plots. Traveling ratios of Pol II were calculated by following a previously described strategy^38^.

*Co-immunoprecipitation (co-IP), mass spectrometry and related analysis*

Co-IP assays were performed as previously described with minor modification^58^. Nuclear extract (NE) from HEL cells was diluted 2 to 3-fold by adding NE dilution buffer or dialyzed against BC-150 buffer to make its salt concentration close to 150 mM. Antibodies were incubated with Dynabeads protein A at 4°C for 3 hours, respectively, and then cross-linked to beads by 25 mM DMA (Pierce, cat. no. 20660) at room temperature for 1 hour. Usually, 0.5 to 1 mg NE was used for each regular co-IP while 75 mg NE was used for each large-scale co-IP for mass spectrometry. After overnight incubation at 4°C, bead-antibody-protein complexes were washed with BC-150 buffer (10 mM Tris-HCl, 0.2 mM EDTA, 150 mM KCl, 20% glycerol, and 0.1% NP-40, pH 7.9) for 4 times and 6 times for regular co-IP and large-scale co-IP, respectively. Additional 2 washes with PBS were added afterwards for large-scale co-IP. The protein complexes were eluted from beads by 50 mM glycine (pH 2.4), immediately neutralized in 1 M Tris-HCl, pH 7.4, and analyzed by Western blot or a Multidimensional Protein Identification Technology (MudPIT) system. Antibodies used for co-IP experiments are listed in Table S2.

MS/MS spectra were searched using MASCOT engine (Matrix Science, London, UK; version 2.2) against the Human UniProt database (downloaded at May 2019; 171145 sequences). For protein identification, the following options were used: Peptide mass tolerance=20 ppm, MS/MS tolerance=0.1 Da, Enzyme=Trypsin, Missed cleavage=2, Fixed modification: Carbamidomethyl (C), Variable modification: Oxidation(M); score > 20. In total, 1,197 and 210 non-redundant proteins were identified in AF9-IP and IgG-IP samples, respectively. Proteins identified in the AF9-IP sample were filtered according to the following criteria: 1) The unique peptide count of a protein in the IgG-IP sample (UPC_IgG_) should be no larger than 1; 2) If the UPC_IgG_ of a protein is 0, the unique peptide count of the same protein in the AF9-IP sample (UPC_AF9_) should be at least 2; and if the UPC_IgG_ of a protein is 1, UPC_AF9_ should be at least 3.667 proteins that met the requirement were referred to as AF9-accosiated proteins.

**Supplementary Tables**

**Table S1. Primers for shRNA and gRNA cloning**

| Name | Sequence | TRC number | Note |
| --- | --- | --- | --- |
| MLL1 shRNA1  Forward | CCGGGCACTGTTAAACATTCCACTTCTCGAGAAGTGGAATGTTTAACAGTGCTTTTTG | TRCN0000234744 | For MLL1 shRNA1 cloning |
| MLL1 shRNA1 Reverse | AATTCAAAAAGCACTGTTAAACATTCCACTTCTCGAGAAGTGGAATGTTTAACAGTGC | TRCN0000234744 | For MLL1 shRNA1 cloning |
| MLL1 shRNA2  Forward | CCGGTGCCAAGCACTGTCGAAATTACTCGAGTAATTTCGACAGTGCTTGGCATTTTTG |  | For MLL1 shRNA2 cloning |
| MLL1 shRNA2 Reverse | AATTCAAAAATGCCAAGCACTGTCGAAATTACTCGAGTAATTTCGACAGTGCTTGGCA |  | For MLL1 shRNA2 cloning |
| AF9 shRNA1  Forward | CCGGTTCAAAGCCTCACAAATTAATCTCGAGATTAATTTGTGAGGCTTTGAATTTTTG |  | For AF9 shRNA1 cloning |
| AF9 shRNA1  Reverse | AATTCAAAAATTCAAAGCCTCACAAATTAATCTCGAGATTAATTTGTGAGGCTTTGAA |  | For AF9 shRNA1 cloning |
| AF9 shRNA2  Forward | CCGGGCACAGTAACATACAGCACTTCTCGAGAAGTGCTGTATGTTACTGTGCTTTTTG | TRCN0000005792 | For AF9 shRNA2 cloning |
| AF9 shRNA2  Reverse | AATTCAAAAAGCACAGTAACATACAGCACTTCTCGAGAAGTGCTGTATGTTACTGTGC | TRCN0000005792 | For AF9 shRNA2 cloning |
| AF9 shRNA3  Forward | CCGGGCCAGTGTGTTACTCATCTTTCTCGAGAAAGATGAGTAACACACTGGCTTTTTG | TRCN0000005790 | For AF9 shRNA3 cloning |
| AF9 shRNA3  Reverse | AATTCAAAAAGCCAGTGTGTTACTCATCTTTCTCGAGAAAGATGAGTAACACACTGGC | TRCN0000005790 | For AF9 shRNA3 cloning |
| AF9 shRNA4  Forward | CCGGGCATACCTAGATGAACTGGTACTCGAGTACCAGTTCATCTAGGTATGCTTTTTG | TRCN0000005793 | For AF9 shRNA4 cloning |
| AF9 shRNA4  Reverse | AATTCAAAAAGCATACCTAGATGAACTGGTACTCGAGTACCAGTTCATCTAGGTATGC | TRCN0000005793 | For AF9 shRNA4 cloning |
| AF9 gRNA  Forward | CACCGAAGTTAGCTTTTCACAGCGG |  | For AF9 gRNA cloning |
| AF9 gRNA  Reverse | AAACCCGCTGTGAAAAGCTAACTTC |  | For AF9 gRNA cloning |

**Table S2. Antibodies for WB, co-IP and CUT&Tag.**

| Target | Vender | Catalogue number | Applications |
| --- | --- | --- | --- |
| AFF1 | Bethyl Laboratories | A302-344A | WB |
| AF9-N | Bethyl Laboratories | A300-595A | WB |
| AF9-N | Bethyl Laboratories | A300-596A | WB, CUT&Tag |
| AF9-C | Diagenode | A300-868A | CUT&Tag |
| CDK9 | Bethyl Laboratories | A303-493A | WB |
| CDK9 | ABclonal | A0886 | CUT&Tag |
| CDK9 | Santa Cruz | sc-13130 | WB |
| MLL1 (456) | E. Canaani Lab |  | co-IP,  CUT&Tag |
| MLL1 (468) | E. Canaani Lab |  | WB, co-IP,  CUT&Tag |
| Pol ll | Santa Cruz | sc-47701 | WB, CUT&Tag |
| Pol ll (Ser-2p) | Millipore | 04-1571 | WB |
| Pol ll (Ser-5p) | Millipore | 04-1572 | WB |
| RBBP5 | Bethyl Laboratories | A300-109A | WB |
| TBP | Santa Cruz | sc-56795 | CUT&Tag |
| Anti-FLAG M2 | Sigma-Aldrich | F1804 | CUT&Tag |
| TFIIEβ | GeneTex | GTX105029 | CUT&Tag |
| β-ACTIN | Santa Cruz | sc-47778 | WB |

**Table S3. Primers for qRT-PCR**

| MLL1 Forward1 | GGTTTGCTTTCTCTGTGCCA | For qRT-PCR of MLL1 |
| --- | --- | --- |
| MLL1 Reverse1 | TAGCCTGATGTTGCCTTCCA | For qRT-PCR of MLL1 |
| MLL1 Forward2 | CGATCAAATGCCCGCCTAAA | For qRT-PCR of MLL1 |
| MLL1 Reverse2 | TCAGGAGGCTGACGATGTTT | For qRT-PCR of MLL1 |
| AF9 Forward1 | GCAGTTCTGCTTCTTCACCC | For qRT-PCR of MLL-AF9 |
| AF9 Reverse1 | GGTTTTGTCCAGCGAGCAAA | For qRT-PCR of MLL-AF9 |
| AF9 Forward2 | CACCCCTACATCACGAACCT | For qRT-PCR of MLL-AF9 |
| AF9 Reverse2 | GTCCAGCGAGCAAAGATCAA | For qRT-PCR of MLL-AF9 |
| β-Actin Forward | TGACGTGGACATCCGCAAAG | Internal control for qRT-PCR |
| β-Actin Reverse | CTGGAAGGTGGACAGCGAGG | Internal control for qRT-PCR |

**Supplementary Figures**


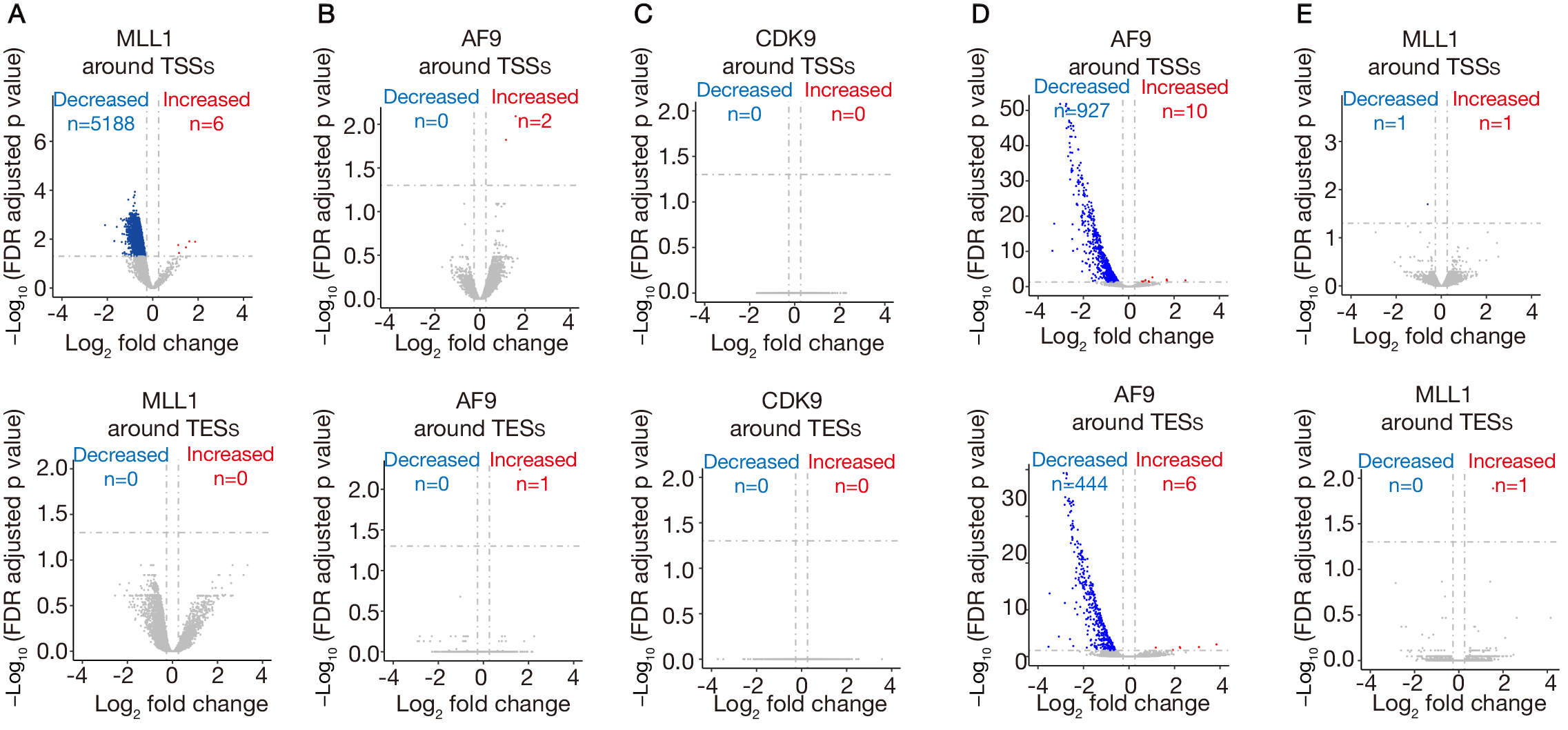


**Supplementary Figure 1. Chromatin occupancy of MLL1 and AF9 is independent of each other in HEL cells.**

*A-C*, Volcano plots showing occupancy changes of MLL1 (*A*), AF9 (*B*) and CDK9 (*C*) on MLL1-bound genes in MLL1 KD cells relative to control cells. *D* and *E*, Volcano plots showing occupancy changes of AF9 (*D*) and MLL1 (*E*) on AF9-bound genes in AF9 KD cells relative to control cells.


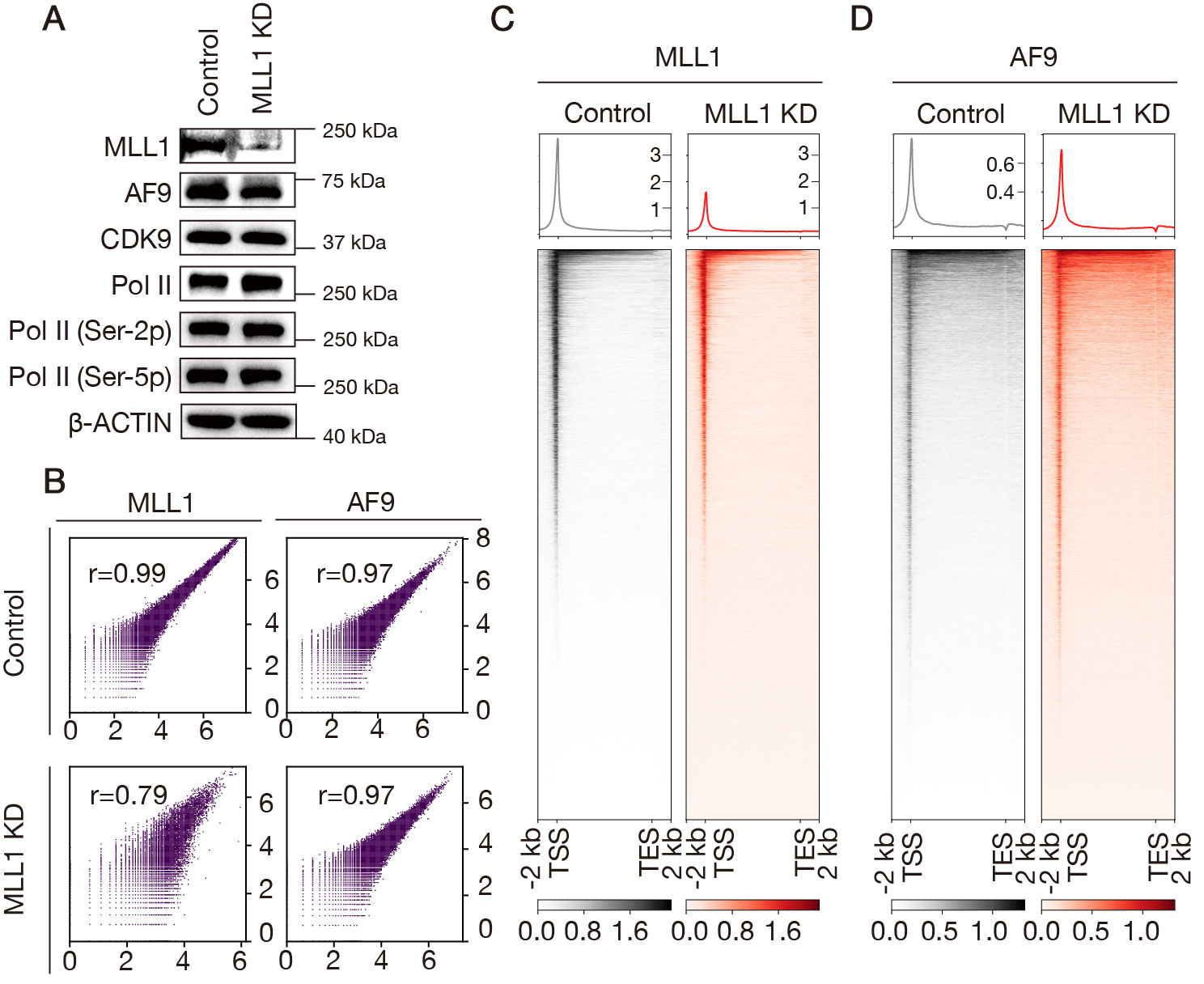


**Supplementary Figure 2. MLL1 knockdown by shRNA#2 minimally affected chromatin occupancy of AF9.**

*A*, Western blot (WB) comparing cellular level of MLL1, AF9, Pol II, Pol II (Ser-2p) and Pol II (Ser-5p) in control and MLL1 KD cells. *B*, Correlation plots for biological replicates of MLL1 and AF9 CUT&Tag experiments (n=2). *C* and *D*, Genome-wide meta-gene profiles and heatmaps of MLL1 (*C*) and AF9 (*D*) CUT&Tag experiments in control and MLL1 KD cells.


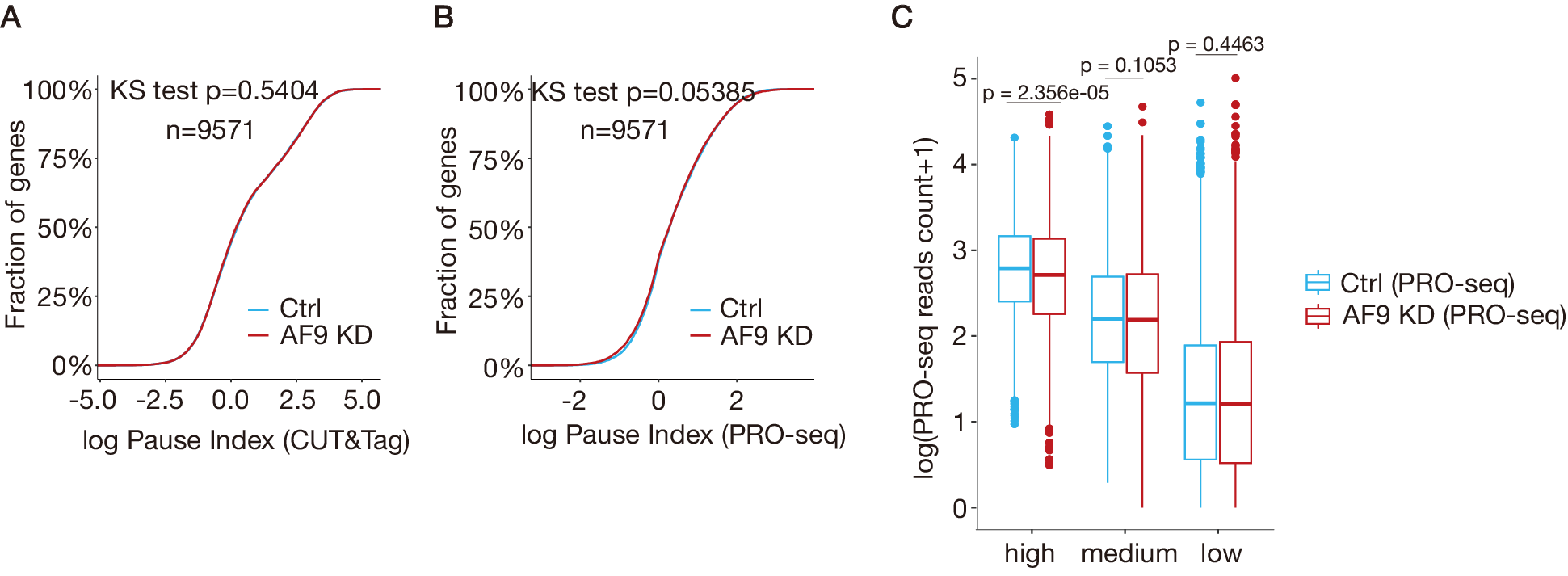


**Supplementary Figure 3. AF9 knockdown affects promoter-proximal pause of a small number of highly paused genes.**

*A*, Comparison of TR of Pol II in control and AF9 KD cells using the total Pol II CUT&Tag data. *B*, Comparison of TR of engaged Pol II in control and AF9 KD cells using the PRO-seq data. *C*, Group-wise comparison of TR of engaged Pol II in control and AF9 KD cells using the PRO-seq data. Genes expressed in control cells were divided into three groups, i.e., highly paused (PI ≥ 3), moderately paused (1.5 ≤ PI < 3) and non-paused (PI < 1.5), and PI of genes in control and AF9 KD cells was compared group-wisely.


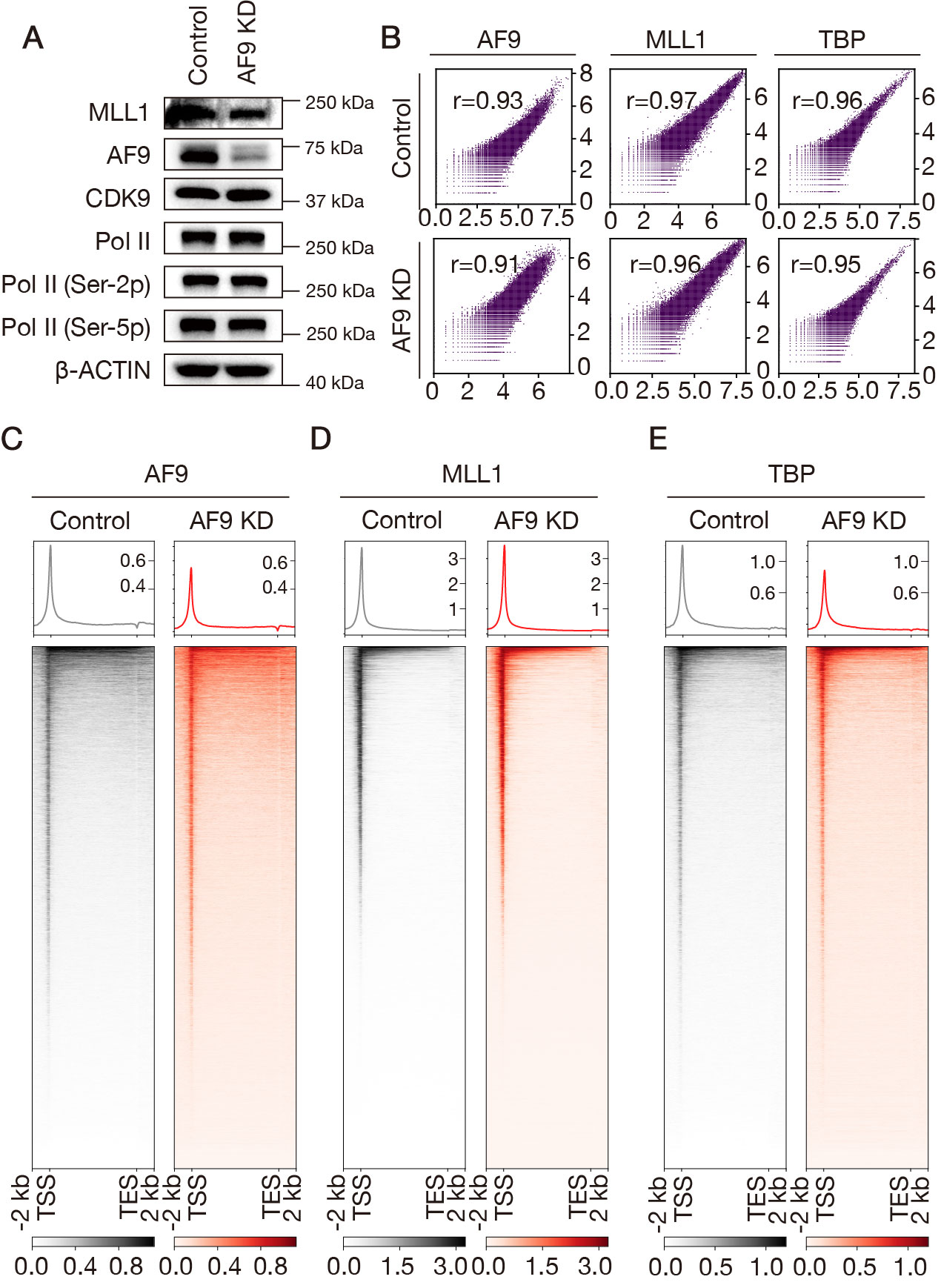


**Supplementary Figure 4. AF9 knockdown by shRNA#2 minimally affected chromatin occupancy of MLL1.**

*A*, Western blot (WB) comparing cellular level of MLL1, AF9, Pol II, Pol II (Ser-2p) and Pol II (Ser-5p) in control and AF9 KD cells. *B*, Correlation plots for biological replicates of AF9, MLL1 and TBP CUT&Tag experiments (n=2). *C, D* and *E*, Genome-wide meta-gene profiles and heatmaps of AF9 (*C*), MLL1 (*D*) and TBP (*E*) CUT&Tag experiments in control and AF9 KD cells.


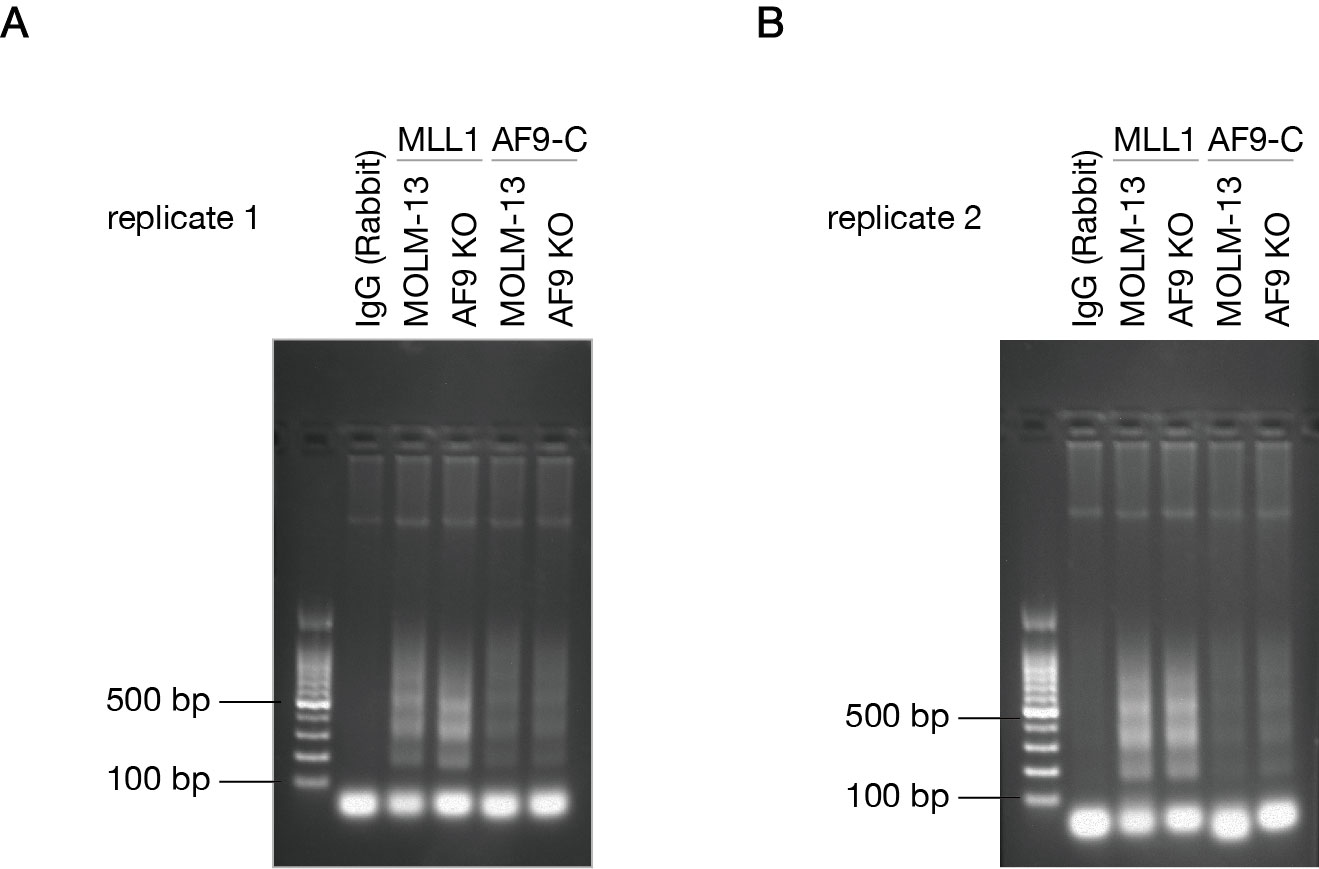


**Supplementary Figure 5. Agarose gel pictures of CUT&Tag experiments in MOLM-13-AF9 KO cells.**

*A* and *B*, Agarose gel pictures of biological replicates 1 (*A*) and 2 (*B*) of MLL1 and MLL-AF9 (using AF9-C antibody) CUT&Tag experiments in control (MOLM-13) and MOLM-13-AF9 KO cells.


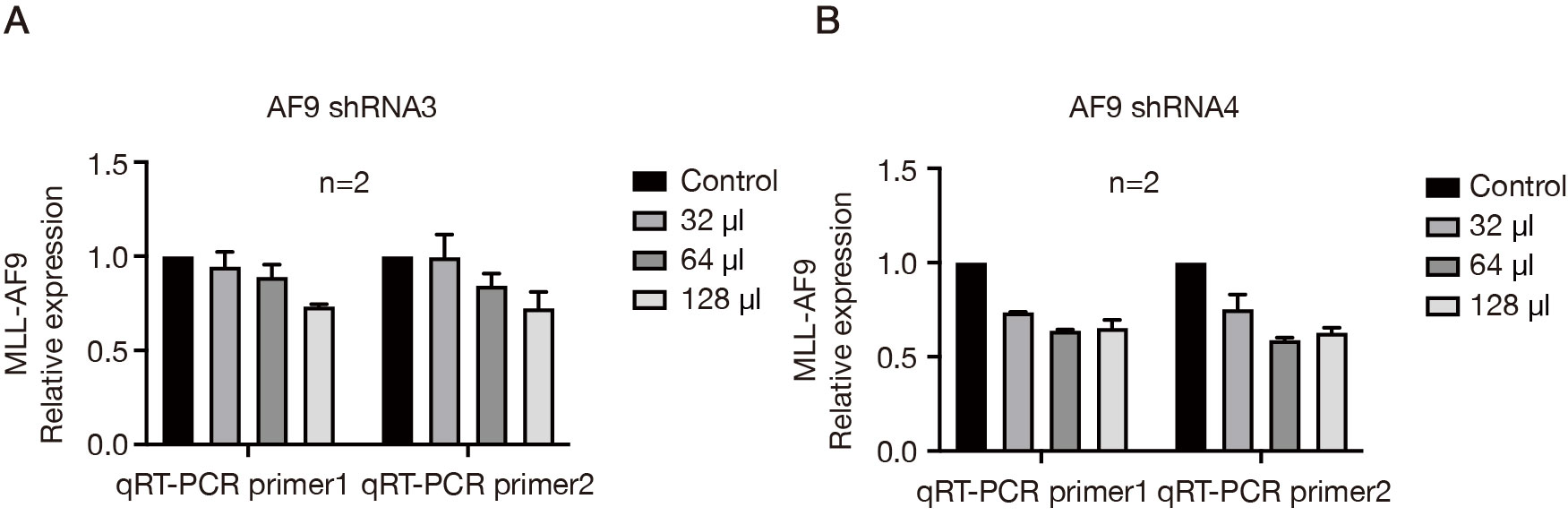


**Supplementary Figure 6. qRT-PCR results of MLL-AF9 knockdown by AF9 shRNAs in MOLM-13-AF9 KO cells.**

*A* and *B*, qRT-PCR results of MLL-AF9 knockdown by different amount of lentiviral AF9 shRNAs #3 (*A*) and #4 (*B*) in MOLM-13-AF9 KO cells.


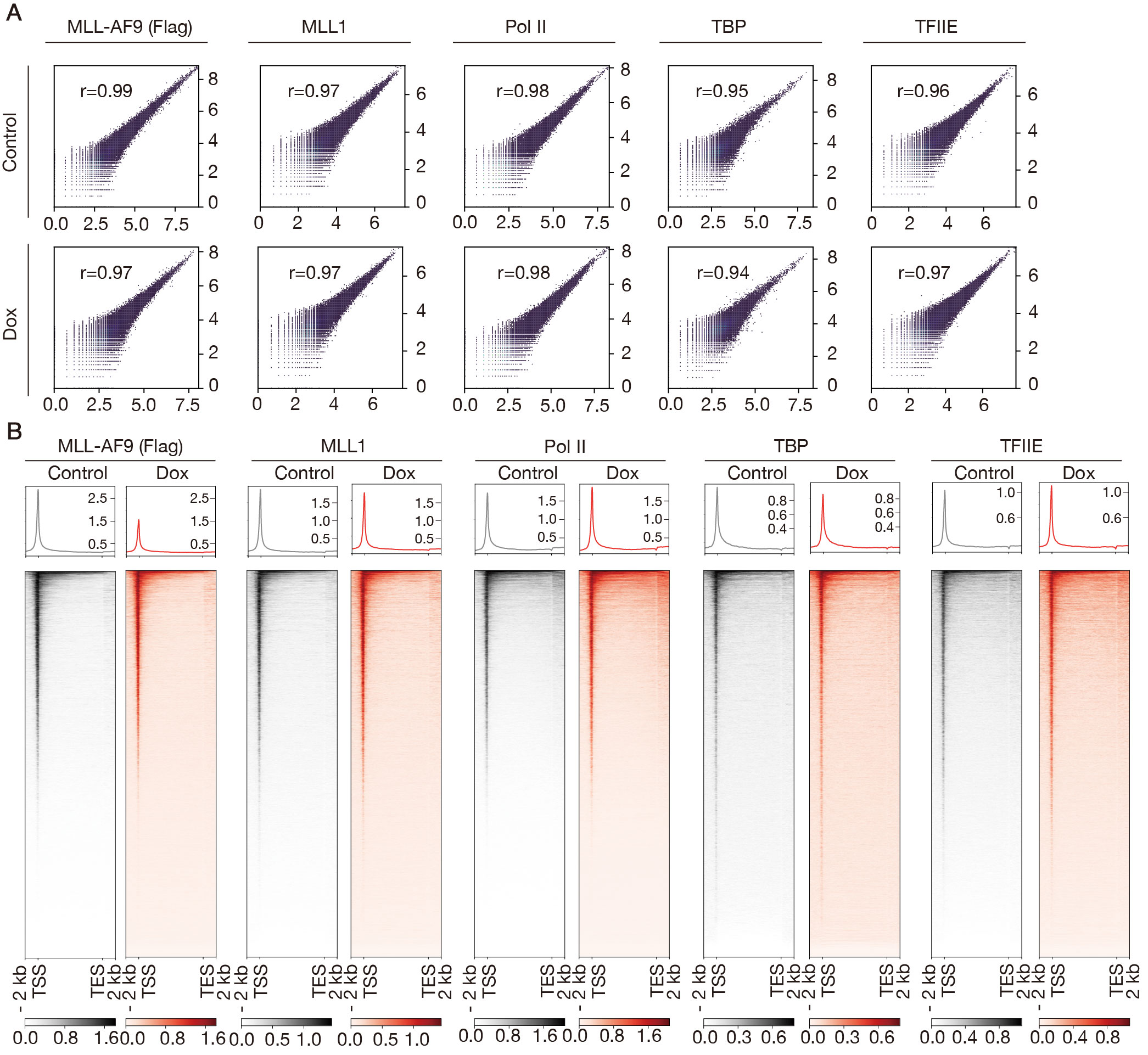


**Supplementary Figure 7. MLL-AF9 downregulation minimally affected global occupancy of MLL1, Pol II, TBP and TFIIE.**

*A*, Correlation plots for biological replicates of CUT&Tag for MLL-AF9, MLL1, Pol II, TBP and TFIIE in untreated and doxycycline (Dox) treated iMA9 cells (n=2). *B*, Genome-wide meta-gene profiles and heatmaps of MLL-AF9, MLL1, Pol II, TBP and TFIIE CUT&Tag experiments in untreated and doxycycline-treated iMA9 cells.
